# Quantifying the Role of Adverse Events in the Mortality Difference between First and Second-Generation Antipsychotics in Older Adults: Systematic Review and Meta-Synthesis

**DOI:** 10.1371/journal.pone.0105376

**Published:** 2014-08-20

**Authors:** John W. Jackson, Sebastian Schneeweiss, Tyler J. VanderWeele, Deborah Blacker

**Affiliations:** 1 Division of Pharmacoepidemiology and Pharmacoeconomics, Department of Medicine, Brigham and Women's Hospital & Harvard Medical School, Boston, Massachusetts, United States of America; 2 Department of Epidemiology, Harvard School of Public Health, Boston, Massachusetts, United States of America; 3 Department of Biostatistics, Harvard School of Public Health, Boston, Massachusetts, United States of America; 4 Gerontology Research Unit, Department of Psychiatry, Massachusetts General Hospital and Harvard Medical School, Boston, Massachusetts, United States of America; University of British Columbia, Canada

## Abstract

**Background:**

Observational studies have reported higher mortality among older adults treated with first-generation antipsychotics (FGAs) versus second-generation antipsychotics (SGAs). A few studies examined risk for medical events, including stroke, ventricular arrhythmia, venous thromboembolism, myocardial infarction, pneumonia, and hip fracture.

**Objectives:**

1) Review robust epidemiologic evidence comparing mortality and medical event risk between FGAs and SGAs in older adults; 2) Quantify how much these medical events explain the observed mortality difference between FGAs and SGAs.

**Data sources:**

Pubmed and Science Citation Index.

**Study eligibility criteria, participants, and interventions:**

Studies of antipsychotic users that: 1) evaluated mortality or medical events specified above; 2) restricted to populations with a mean age of 65 years or older 3) compared FGAs to SGAs, or both to a non-user group; (4) employed a “new user” design; (5) adjusted for confounders assessed prior to antipsychotic initiation; (6) and did not require survival after antipsychotic initiation. A separate search was performed for mortality estimates associated with the specified medical events.

**Study appraisal and synthesis methods:**

For each medical event, we used a non-parametric model to estimate lower and upper bounds for the proportion of the mortality difference—comparing FGAs to SGAs—mediated by their difference in risk for the medical event.

**Results:**

We provide a brief, updated summary of the included studies and the biological plausibility of these mechanisms. Of the 1122 unique citations retrieved, we reviewed 20 observational cohort studies that reported 28 associations. We identified hip fracture, stroke, myocardial infarction, and ventricular arrhythmias as potential intermediaries on the causal pathway from antipsychotic type to death. However, these events did not appear to explain the entire mortality difference.

**Conclusions:**

The current literature suggests that hip fracture, stroke, myocardial infarction, and ventricular arrhythmias partially explain the mortality difference between SGAs and FGAs.

## Introduction

In 2008, nearly 3 million U.S. adults age 65 or older received prescriptions for antipsychotic medications, 63% of which involved off-label use not approved by the U.S. Food and Drug Administration (FDA) [1]. One common form of such off-label use targets agitation, aggressiveness, and psychosis in dementia patients, which can disrupt medical and institutional care. The decision to treat older adults with an antipsychotic involves a careful trade-off between clinical benefit and risk for serious adverse events [2]. In randomized controlled trials of dementia patients, second-generation antipsychotic agents (SGAs) increased mortality by as much as 54% over placebo in the first 10–12 weeks following initiation [3], leading the FDA in 2005 to issue Black Box warnings about their excess mortality in 2005. Subsequent observational studies demonstrated even higher mortality during the first 24 weeks) after initiation with first-generation agents (FGAs), leading to Black Box warnings for FGAs in 2008. While SGAs currently represent the vast majority of off-label use [1], FGAs represent two to 20% of the antipsychotics prescribed in U.S. nursing homes, a variation that may be driven by differences in institutional prescribing culture and the lower cost of FGAs [4]. As of 2008, all FGAs approved for use in the U.S. were available as generics and cost $9–$26 per prescription, whereas only two of the seven approved SGAs were available as generics and cost $85–$345 per prescription [1].

It is not clear why FGAs increase mortality more than SGAs in older adults soon after they begin therapy. Randomized efficacy and so-called pragmatic or effectiveness trials are usually not designed to answer this question. Observational studies reported higher mortality with FGAs for cerebrovascular and respiratory causes [5], [6], but these categories are broad and may suffer from misclassification [7]–[9]. Other studies have investigated the association between antipsychotic type (FGA versus SGA) and the risk of medical events, such as stroke, ventricular arrhythmia, venous thromboembolism, myocardial infarction, hip fracture, and pneumonia [10]–[18]. It is unclear from these studies' relative risk estimates alone which medical events are major contributors to the mortality difference between FGAs and SGAs. Furthermore, recent systematic reviews of these studies share this same limitation and they have typically been limited to one medical event [19]–[27].

To better understand differences in FGA and SGA related mortality in older adults, we sought to make two contributions to the literature. First, we provide an updated, systematic review of the epidemiologic evidence comparing FGA and SGA risk among older adults for mortality, stroke, ventricular arrhythmia, venous thromboembolism, myocardial infarction, hip fracture, and pneumonia. Unlike previous reviews that compared the risk for various medical events between FGAs and SGAs, we only included studies whose design and analysis choices were relevant for evaluating short-term effects of medications. Second, we used epidemiologic data from the included studies, along with published mortality rates, to quantify how much of the differential mortality between FGAs and SGAs is potentially mediated by their differences in risk for these medical events. Such data goes beyond describing the relative risk for medical events to quantitatively consider their frequency and lethality, which may be helpful when updating monitoring guidelines.

## Methods

### Systematic review of the relationship between type of antipsychotic use (FGA versus SGA) and risk of mortality or medical events

We report this systematic review according to the standards outlined in the PRISMA statement [28] (see Checklist S1). The design of this study evolved from a narrative review to a systematic and quantitative meta-synthesis of data from various sources, which is reported here. The meta-synthesis aimed to explain mortality differences at six-months to remain consistent with previous literature (nine out of the 12 identified studies reporting mortality data did so for six months follow-up; five studies evaluated 40 days or less follow-up, and two examined mortality after one year of follow-up). Further detail on the rationale, extracted data, lists of obtained studies, and bias analyses are provided in the appendix (File S3, S5, S6, and S7).

#### Search

We searched Pubmed through October 9, 2012 for epidemiologic studies reporting the risk of mortality, stroke, ventricular arrhythmias, sudden cardiac death, venous thromboembolism, myocardial infarction, pneumonia, and hip fracture in older antipsychotic users. These events were chosen apriori through an initial review of the literature. Our final search strategy included free-text and controlled vocabulary terms (e.g. Medical Subject Headings) for these topics, their synonyms, abbreviations, and alternate spellings (see File S1).

#### Inclusion and exclusion criteria

For this review, our inclusion criteria applied to studies evaluating the risk for medical events in FGA and SGA users. Our search strategy included finding comparative randomized trials, but we did not find any that reported on the medical events of interest as an outcome (listed in criterion #1). Thus, we formulated criteria to reduce the potential for bias in observational studies' estimates of the relationship between antipsychotic type (FGA versus SGA) and mortality or medical events, and included observational studies with the following characteristics: (1) evaluated antipsychotic users' risk of mortality or the following medical events: stroke, ventricular arrhythmia or sudden cardiac death, venous thromboembolism, pneumonia, or hip fracture (2) directly compared FGAs to SGAs or compared both to a non-user reference group; (3) the mean age of the study population was 65 or greater or age-stratified results—absolute rates or relative risks—were provided for adults over age 65; (4) the study sample was restricted to “new users” of antipsychotic medications or required a washout-period of no use prior to cohort entry; (5) adjusted for potential confounders that were assessed prior to antipsychotic initiation; (6) and did not require a minimum period of survival after antipsychotic initiation for inclusion in the analysis. We required new-user designs because prior studies demonstrate that the mortality hazard is highest immediately after antipsychotic initiation and decreases thereafter [29]. For the same reason, we excluded studies that required a minimum period of survival after antipsychotic initiation for cohort entry. We only considered studies where covariates were assessed prior to antipsychotic initiation because adjusting for subsequent changes in health does not control for confounding and may increase bias [30]. The rationale for these criteria is discussed further in the appendix (see File S3).

#### Selection process

**Figure 1:**
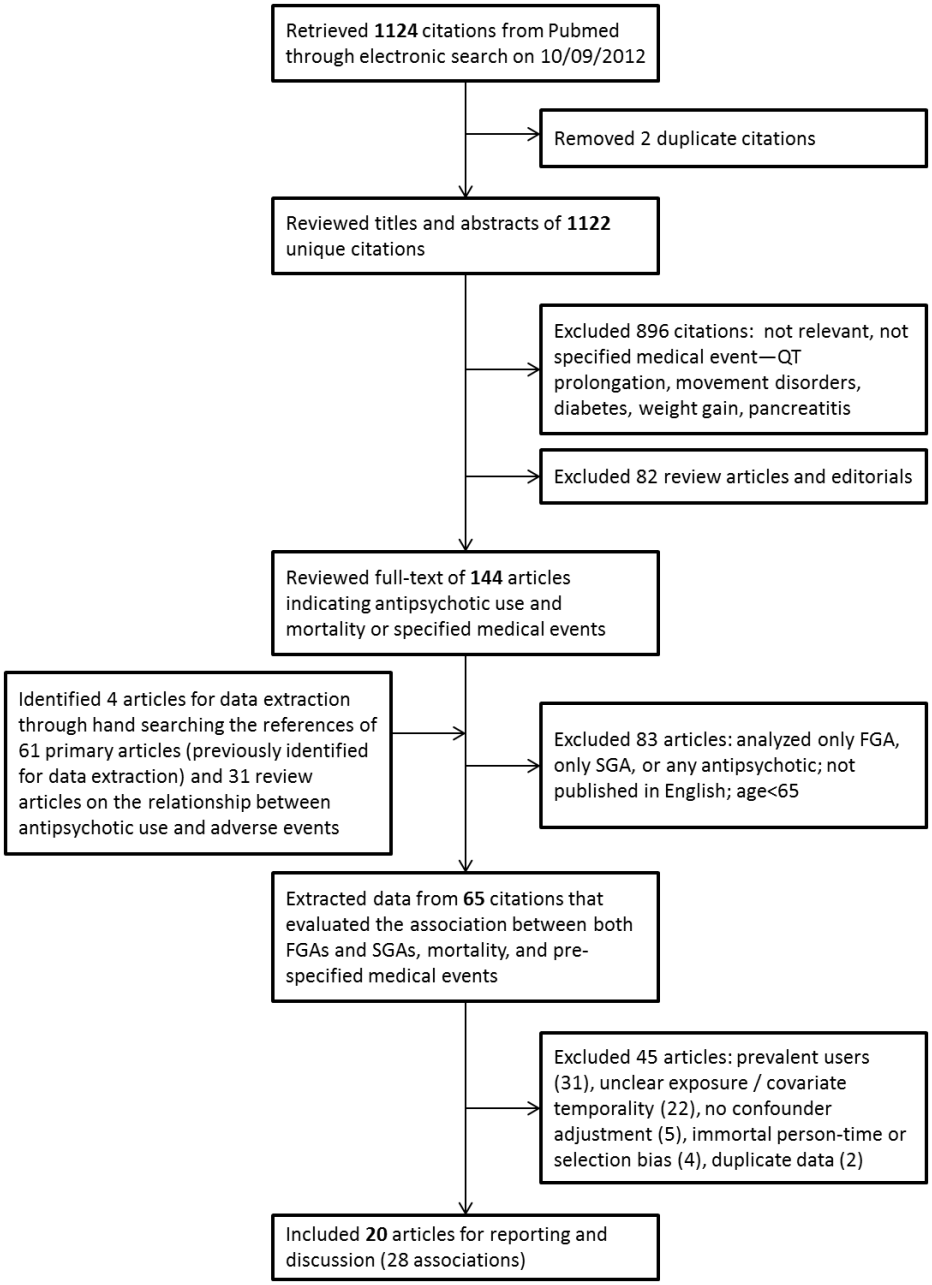
Flowchart for search execution.

We reviewed the titles and abstracts of articles retrieved from Pubmed and selected primary research articles that met criterion 1. From those articles that then met criterion 2 and were published in English, we extracted information about their study population, design, outcome assessment and occurrence, and adjusted results. To locate additional articles for data extraction, Science Citation Index was used to “hand search” the titles and abstracts of references cited by these articles (and those cited by relevant review articles identified earlier during the selection process). Multiple studies reporting results from the same administrative records were included when differences in study design had potential for describing mortality or medical event risk in clinically relevant subgroups (e.g. nursing home and dementia), populations (e.g. veterans), for use over different calendar periods or length of follow-up, or when the analyses adjusted for different sets of potential confounders. The studies that met criteria 3 through 6 were included in our review (see Figure 1 for flowchart). A list of the excluded articles is provided for each medical event in the appendix (see File S7). The article selection process and data extraction were carried out by JWJ.

### Estimation of mortality rates for medical events

To obtain estimates of six-month mortality associated with each medical event, we also searched Pubmed for articles reporting the pre-hospital, in-hospital, and/or post-discharge mortality (for follow-up of one year or less) associated with stroke, ventricular arrhythmia, acute myocardial infarction, venous thromboembolism, hip fracture, and pneumonia. When possible, we chose review articles that summarized the evidence on mortality for the medical event, and otherwise chose recently published articles with the broadest possible study population in the appropriate age range, and avoided those that focused on a specific disease group or clinical profile. None of these articles provided enough data to precisely calculate the six-month mortality due to the medical event for persons in this age range; several studies were not limited to persons over age 65, did not provide rates for pre-hospital mortality, reported a wide range of estimates, or only reported mortality rates after discharge from an inpatient stay. We chose a plausible estimate halfway between the total mortality for the given medical event at three months and one year, when available. When the mortality was reported separately for medical event subtypes, we took a weighted average according to the frequency of the medical event subtypes. If the studies included persons younger than age 65 or did not report out-of-hospital deaths, we chose the total mortality at one year. If a range for six-month mortality was reported, we chose a plausible estimate near the upper bound (in the section that follows, this choice serves to provide an upper bound for the proportion mediated). Using these approaches, we made our best approximation to the nearest 5% on the risk scale.

To obtain estimates for the mortality among persons not experiencing a particular medical event, we estimated the six-month mortality as half the annual age-standardized mortality among persons aged 65 years or older from mortality tables published by the U.S. National Center for Health Statistics [31].

### Synthesizing the evidence for the contribution of medical events to differences in mortality between FGA and SGA users

We calculated the difference in mortality, comparing FGAs to SGAs, mediated by their difference in risk for a particular medical event (stroke, for example) after antipsychotic initiation using the following model [32]:
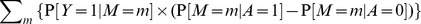



where *A* represents the type of antipsychotic initiated {1 = FGA, 0 = SGA}, *M* represents the occurrence of the medical event after antipsychotic initiation {1 = occurs, 0 = does not occur}, and *Y* represents mortality during six months follow-up {1 = death, 0 = survival} [33]. This model projects the difference in mortality between FGA and SGA users who do and do not experience the medical event after antipsychotic initiation. To obtain the proportion of the mortality difference that is mediated by the medical event, we divided this quantity by the overall difference in mortality, 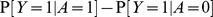. The risk of the medical event in SGA users, 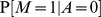, was calculated from the included studies as the average medical event rate (among SGAs) per 50 person-years to approximate six-month risk; most of these rates were originally reported in units of 100 or 1000 person-years. Similarly 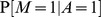 represents the average medical event rate among FGAs and was calculated as the product between the average medical event rate among SGAs and the average adjusted six-month relative risk for the medical event comparing FGAs and SGAs (see File S4 for more detail). 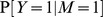 represents the six-month mortality given the medical event occurs, and 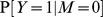 is the six-month mortality given the medical event does not occur. 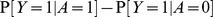 was taken as the smallest difference in mortality comparing FGAs to SGAs from studies that reported this quantity (to provide an upper bound for the proportion mediated). Examining the model, we see that the projected mortality due to the medical event (and thus the proportion mediated) depend on the absolute occurrence of the medical event among SGAs, the difference in risk for the medical event between FGAs and SGAs, and the difference in mortality for those who do and do not experience the medical event. The projected mortality difference for a given medical event (and the proportion mediated) uses summary data from the reviewed studies and published mortality data (a table describing the model components, source data and populations used to estimate them is provided in the appendix (see File S4). In this application, the model requires an assumption that the estimates for excess mortality 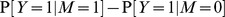, which are based on older adults in clinical and population studies, apply to older adults treated with antipsychotics. We address the implications of this and other limitations in the Discussion.

### Bounds and bias analysis

The studies that yield data on the medical event rate 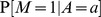 rely on diagnostic records in claims data which typically have poor sensitivity. Also, the six-month mortality estimates were interpolated from pre-hospital, 30-day and one-year estimates in the published literature. These values were used to obtain point-estimates for the proportion mediated. To provide bounds that reflect these potential sources of error, we re-estimated the proportion mediated by applying plausible values for the sensitivity of diagnostic algorithms used across studies. We arbitrarily chose a sensitivity of 0.9 for hip fracture because it requires hospitalization and should be well-captured in claims data, 0.2 for ventricular arrhythmia because it often results in sudden death before hospitalization is possible, and 0.5 for other medical events that can lead to pre-hospital death (e.g. stroke and myocardial infarction). We also incorporated plausible minimum and maximum values for excess mortality. These were taken directly as the ranges for six-month mortality reported in the source studies; when these were not available we calculated the maximum plausible value for excess mortality as the one-year medical event mortality minus the 30-day general population mortality, and calculated the minimum value as the 30-day medical event mortality minus the one-year general population mortality. We also carried out formal bias analyses to further examine how more extreme bias scenarios would affect the proportion mediated. Last, as a confirmatory analysis, we repeated our analysis using an alternate method that calculates the proportion mediated within each study and averages across these results. Assumptions, estimation procedures, and results for these bias analyses are provided in the appendix (see File S5 and S6). The lower and upper bounds accounting for potential biases are reported along with point estimates; note that because the bounds take into account poor diagnostic sensitivity while the point estimates do not, in some cases this will result in the lower bound exceeding the point estimate.

### Review of epidemiologic evidence and biological plausibility

For mortality, we summarized the absolute and relative difference in mortality reported by the included studies. For each medical event, we first present the proportion of the mortality difference explained, followed by a brief review of the epidemiologic evidence and biological plausibility. Because our review sought to understand which medical events ultimately contribute to mortality soon after starting an antipsychotic, we focused on the first six months after antipsychotic initiation.

## Results

### Search

We retrieved 1122 unique articles from Pubmed, 63 of which compared the risk of the medical events of interest in FGAs and SGAs. Of these, 20 articles met all inclusion criteria and reported 28 associations between antipsychotic type, mortality, or medical events (Figure 1 and Table 1). These studies were carried out in six distinct data sources from U.S. Medicare and Medicaid, the U.S. and Australian Veterans Affairs health systems, Canadian administrative health records from Ontario and British Columbia, administrative data from a large regional hospital in Hong-Kong, and primary care records from Italy. A list of included and excluded citations for each medical event is provided in the appendix (See File S7).

**Table 1:** Study design characteristics of included and excluded studies by type of event.

All Articles (n = 63)		Included Articles (n = 20)				Excluded Articles (n = 43)							
Reported Associations		ll	FGA > SGA	FGA < SGA	FGA≈ SGA	Il	Prevalent User	Temporality Unclear†	Selection/immortal bias	No confounding adjustment	FGA > SGA	FGA < SGA	FGA≈ SGA
All-Cause Mortality	**19**	**12**	11	0	1	**7**	5	5	1	0	6	2	0
Myocardial Infarction	**5**	**2**	2	0	0	**3**	1	2	1	0	2	1	0
Ventricular Arrhythmia	**6**	**2**	1	0	1	**4**	3	1	1	0	3	0	1
Stroke	**16**	**6**	3	1	2	**10**	7	4	1	2	5	2	3
Hip Fracture	**12**	**2**	2	0	0	**10**	7	5	1	1	3	7	0
Pneumonia	**7**	**3**	0	0	3	**4**	2	2	0	1	0	1	3
Venous Thromboembolism	**9**	**1**	0	1	0	**8**	6	3	1	1	1	2	5
All events	**74**	**28**	19	2	7	**46**	32	23	6	5	20	15	12

† unclear if covariates were assessed before antipsychotic initiation.

### Overall summary of results

**Figure 2:**
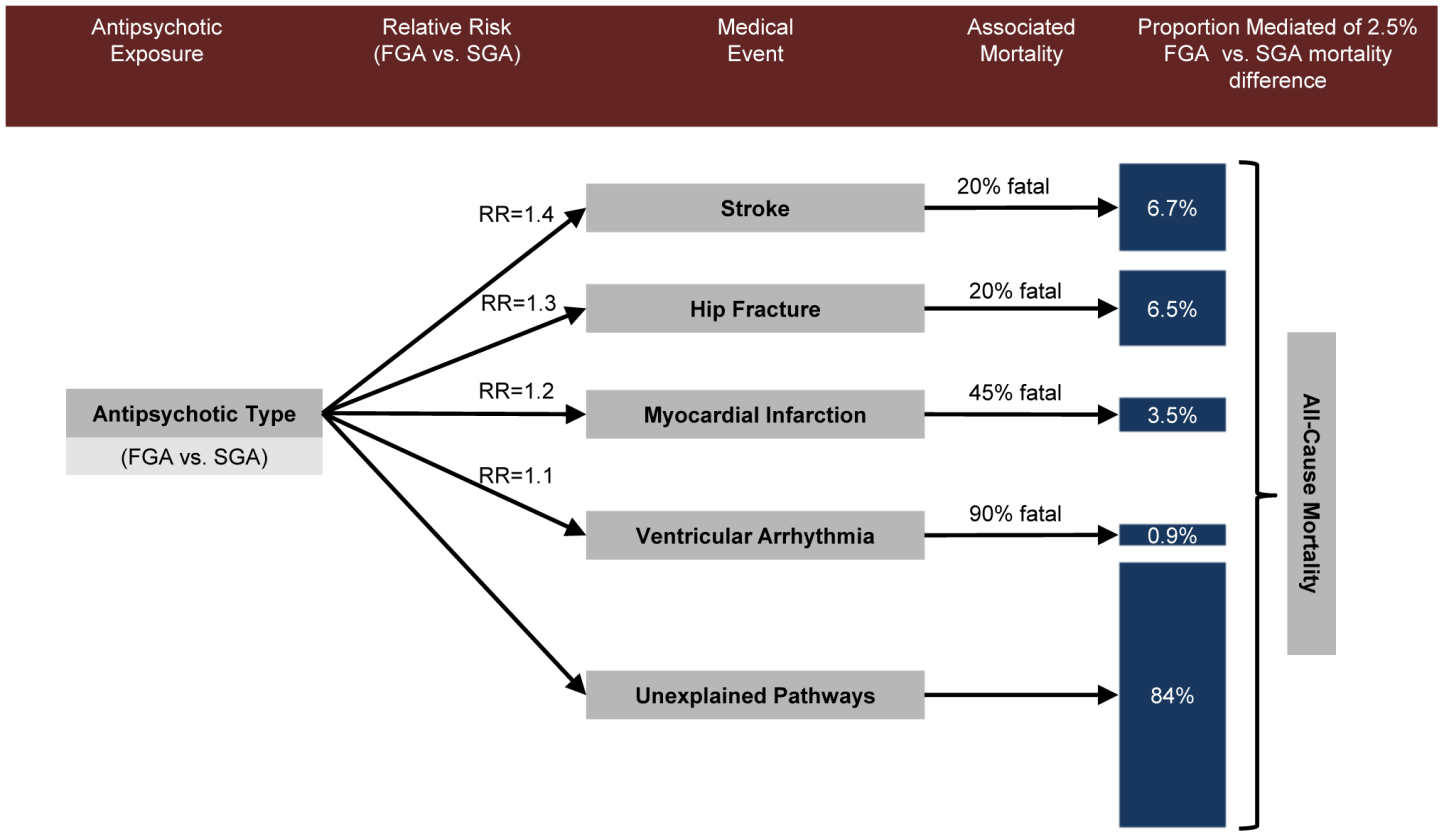
The causal pathway from antipsychotic type to mortality through medical events. The average medical event rates (per 100 person-years) were 4.7 for stroke, 0.48 for ventricular arrhythmia, 1.2 for venous thromboembolism, 2.0 for myocardial infarction, 6.2 for hip fracture, and 4.8 for pneumonia. In calculations they were scaled to units of 50-person years to approximate six-month risk.

Among the included studies, we found higher mortality for FGAs than SGAs in the first six months after starting antipsychotic therapy (average relative risk = 1.4; average risk difference = 4.3%, ranging from 2.5% to 7.3% in samples containing community-dwelling and long-term care residents). Table 2 describes the characteristics of each included study: the study population, design, mortality and medical event rates, and relative risks comparing FGAs to SGAs. Table 3 presents the values (obtained by averaging across study-level data) used to estimate the projected mortality difference for each medical event. These projections were divided by the smallest observed total effect (2.5%) to obtain the proportion mediated by each medical event (so that the proportion mediated estimates reflect an upper limit absent other biases; see File S6 for results using the largest observed total effect (7.3%). Figure 2 depicts the causal pathway from antipsychotic type to mortality, showing the intermediate medical events for which FGAs carry greater risk than SGAs. Based on our model, up to 6.7% of the higher mortality for FGAs was due to stroke, 6.6% to hip fracture, 3.5% to myocardial infarction, and 0.9% to ventricular arrhythmia (17.4% combined). The lower and upper bounds that adjust for poor diagnostic sensitivity and other potential biases were 7.4% and 18.9% for stroke, 1.3% and 9.2% for hip fracture, 4.2% and 9.5% for myocardial infarction, and 3.9% and 4.8% for ventricular arrhythmia (16.8% and 42.4% combined); the lower bounds are higher than the point estimate because poor sensitivity of diagnostic algorithms leads to downwards bias. With the exception of ventricular arrhythmia, these bounds would be much wider in cases of extreme bias (see File S5 and S6). In the following sections we review the epidemiologic evidence for the differential mortality between FGAs and SGAs, the biological plausibility and differential risk for each medical event, and the mortality associated with each medical event.

**Table 2:** Descriptions of included studies (all conducted and analyzed as cohort studies).

Study, Year	Population	Data Source	Exposure definition and comparison	Outcome	Follow-up	Occurrence in *SGA, Risk (%) Rate (per 100 PY)*	Adjusted Association (95% Cl) *HR = Hazard Ratio, OR = Odds Ratio, RD = Risk Difference, RR = Rate Ratio*
Wang 2005 [34]	22,890 residents, Pennsylvania, mean age = 83	Pharmacy-linked Medicare claims	Use at baseline (FGA vs. SGA)	Death	180 days:	Risk = 14.6	HR = 1.37 (1.27 to 1.49); RD = 7.3% (2.0 to 12.6)
Gill, 2007 [35]	14,213 residents with dementia, Ontario, mean age = 82	Province-wide administrative health records	Use until discontinuation* (FGA vs. SGA)	Death	180 days:	Risk = 14.4	HR = 1.23 (1.00 to 1.50); RD = 2.6% (0.5 to 4.5)**
Kales, 2007 [41]	4,352 beneficiaries with dementia, USA, mean age = 79	Veterans Affairs Administrative health records	Use at baseline (FGA vs. SGA)	Death	365 days:	Risk = 22.6	HR = 0.93 (0.75 to 1.16)
Schneeweiss, 2007 [29]	37,241 residents, British Columbia, mean age = 80	Province-wide administrative health records	Use at baseline (FGA vs. SGA)	Death	180 days:	Risk = 9.6	HR = 1.32 (1.23 to 1.42); RD = 3.5% (2.7 to 4.3)
Liperoti, 2009 [38]	9,729 nursing home residents with dementia, USA, mean age = 84	Medicare claims, SAGE†, MDS‡	Use at baseline (FGA vs. SGA)	Death	180 days:	Rate = 40	HR = 1.41 (1.13 to 1.42)
Pratt, 2010 [36]	16,539 beneficiaries, Australia, mean age = 83	Veterans Affairs Administrative health records	Use at baseline (FGA vs. SGA)	Death	365 days:	Risk = 29.5	RD: 10.6% (9.2 to 12.1)
Rossom, 2010 [39]	9,878 patients with dementia, Texas, mean age = 78	Veterans Affairs Administrative health records	Use until discontinuation* (compound specific vs. non-users)	Death	30 days:	Risk = 1.8	Haloperidol: HR = 2.2 (1.7 to 2.9)
							Olanzapine: HR = 1.3 (1.0 to 1.7)
							Quetiapine: HR = 0.8 (0.6 to 1.1)
							Risperidone: HR = 1.2 (1.0 to 1.4)
Huybrechts, 2011 [42]	82,012 nursing home residents, USA, mean age = 83	Medicare claims, OSCAR§, MDS‡	Use at baseline (FGA vs. SGA)	Death	180 days:	Risk = 19.6	HR = 1.54 (1.46 to 1.61); RD = 8.3% (7.3 to 9.2)
Huybrechts, 2012 [6]	11,445 nursing home residents, USA, mean age = 84	Medicare claims, MDS‡	Use until discontinuation* (haloperidol vs. risperidone)	Death (non-cancer)	180 days:	Rate = 58	Chlorpromazine-equiv. dose >50 mg: HR = 1.70 (1.40 to 2.06)
							Chlorpromazine-equiv. dose ≤50 mg: HR = 1.41 (1.18 to 1.69)
Huybrechts, 2011 [17]	3,844 new nursing home residents, British Columbia, mean age = 84	Province-wide administrative health records	Use at baseline and until discontinuation* (FGA vs. SGA)	Death (non-cancer)	180 days:	Rate = 27	Use at baseline: HR = 1.24 (1.00 to 1.53)
							Until discontinuation*: HR = 1.33 (0.99 to 1.77)
				Pneumonia	180 days:	Rate = 7.6	Use at baseline: HR = 0.96 (0.66 to 1.69)
							Until discontinuation*: HR = 1.03 (0.62 to 1.69)
				Hip Fracture	180 days:	Rate = 8.5	Use at baseline: HR = 1.11 (0.60 to 2.05)
							Until discontinuation*: HR = 1.67 (1.03 to 2.71)
Aparasu, 2012 [43]	3,609 matched pair nursing home residents, USA, mean age = 83	Medicare and Medicaid claims	Use at baseline (FGA vs. SGA)	Death	<40 days:	Not reported	HR = 1.81 (1.49 to 2.18)
					180 days:	Risk = 18.4	HR = 1.41 (1.27 to 1.57)
Kales, 2012 [40]	20,927 beneficiaries with dementia, USA, mean age≥65	Veterans Affairs Administrative health records	Use at baseline (haloperidol vs. risperidone)	Death	180 days:	Rate = 46	Use at baseline: HR = 1.50 (1.35 to 1.67)
							Until discontinuation*: HR = 1.59 (1.36 to 1.85)
Finkel, 2005 [10]	9,545 dementia patients, USA, mean age = 81	Medicaid claims	Use at baseline (haloperidol vs. risperidone) adjusted for duration of use	Cerebrovascular event	90 days:	Risk = 0.87	OR = 1.91 (1.02 to 3.60)
Gill, 2005 [11]	32,710 residents with dementia, Ontario, mean age = 83	Province-wide administrative health records	Use until discontinuation* (FGA vs. SGA)	Ischemic stroke	238 days: (mean)	Rate: 2.6	HR = 1.01 (0.81 to 1.26)
Wang, 2007 [13]	22,890 residents, Pennsylvania, mean age = 83	Pharmacy-linked Medicare claims	Use at baseline (FGA vs. SGA)	Cerebrovascular event/Transient Ischemic Attack	120 days:	Not reported	HR = 1.09 (1.02 to 1.16)
				Ventricular Arrhythmia	120 days:	Not reported	HR = 1.06 (0.96 to 1.17)
				Myocardial Infarction	120 days:	Not reported	HR = 1.16 (0.91 to 1.48)
				Pneumonia	120 days:	Not reported	HR = 0.84 (0.66 to 1.05)
Sacchetti, 2008 [14]	2,255 primary care patients, Italy, mean age = 76	Administrative health records	Use until discontinuation* (FGA vs. SGA)	Stroke	87-112 days: (mean)	Rate = 4.7	Butyrophenones: HR = 1.44 (0.55 to 3.76)
							Phenothiazines: HR = 2.34 (1.01 to 5.41)
Chan, 2010 [16]	1,089 new dementia patients, Hong Kong, mean age = 80	Administrative hospital records	Use until discontinuation* (FGA or SGA vs. non-users)	Cerebrovascular event/Transient Ischemic Attack	867 days: (mean)	Rate = 4.9	FGA: 0.96 (0.58 to 1.59)
							SGA: 1.04 (0.35 to 3.07)
Huybrechts, 2012 [18]	83,959 nursing home residents, USA, mean age = 82	Medicare claims, OSCAR§, MDS‡	Use until discontinuation* (FGA vs. SGA)	Cerebrovascular event/Transient Ischemic Attack	180 days:	Rate = 9.2	HR = 0.81 (0.65 to 1.01)
				Myocardial Infarction	180 days:	Rate = 2.0	HR = 1.23 (0.82 to 1.82)
				Pneumonia	180 days:	Rate = 1.9	HR = 1.28 (0.87 to 1.88)
				Hip Fracture	180 days:	Rate = 3.8	HR = 1.27 (0.94 to 1.72)
Ray, 2009 [15]	276,907 beneficiaries, Tennessee, mean age = 45.7	Medicaid claims and death certificate data	Time-varying use (FGA or SGA vs. non-users)	Sudden cardiac death	803 to 1059 days (median)	Rate = 0.48***	FGA: RR = 1.74 (1.14 to 2.67)
							SGA: RR = 1.86 (1.35 to 2.57)
Liperoti, 2005 [12]	132,018 nursing home residents, USA, age≥65	Medicare claims, SAGE†, MDS‡	Use at baseline (FGA or SGA vs. non-users)	Venous Thromboembolism	180 days:	Rate = 1.24	FGA: HR = 1.02 (0.67 to 1.55)
							SGA: HR = 2.01 (1.50 to 2.70)

*discontinuation of initial antipsychotic or initiation of comparator antipsychotic.

† SAGE: Systematic Assessment of Geriatric Drug Use via Epidemiology database.

‡ MDS: Minimum Dataset baseline and follow-up clinical assessments.

§ OSCAR: Online Survey, Certification and Reporting for U.S.

**This estimate for was for the community-dwelling cohort; the corresponding estimate for the long-term care cohort was RD = 2.2 (95%CI 0.0 to 4.4). We took a weighted average of these estimates according to the reported distribution of community-dwelling and long-term care residents and obtained a point estimate of RD = 2.47%.

***Rate among persons 70 to 74 years of age.

**Table 3:** Medical event occurrence, association with antipsychotic type (FGA vs. SGA), and difference in mortality between FGAs and SGAs due to their difference in risk for the medical event.

Event	Average Medical Event Rate and Range* among SGA users (per 100 PY†)	Average Medical Event Relative Risk and Range* (FGA vs. SGA users)	Six-month Mortality for the Medical Event	Difference in mortality due to differences in medical event risk between FGA and SGA users (lower bound, upper bound accounting for potential bias)**
Stroke	4.7 (2.6 to 9.2)	1.4 (0.81 to 1.91)	20%	0.17% (0.18 to 0.47)
Ventricular Arrhythmia	0.48‡	1.1‡	90%	0.02% (0.10 to 0.12)
Venous Thromboembolism	1.2‡	0.5‡	15%	−0.06% (------- to -------)
Myocardial Infarction	2.0‡	1.2 (1.16 to 1.23)	45%	0.09% (0.10 to 0.24)
Hip Fracture	6.2 (3.8 to 8.5)	1.3 (1.27 to 1.39)	20%	0.16% (0.03 to 0.23)
Pneumonia	4.8 (1.9 to 7.6)	1.0§ (0.84 to 1.28)	20%	0.00% (------- to -------)

*arithmetic average (minimum, maximum) of reported estimates from included studies (as described in the methods section).

† Rates here are shown in units of 100 person-years; in calculations and in the text they were scaled to units of 50 person-years to approximate six-month risk.

‡ Only 1 study contributed to these rounded estimates (RR = 1.06 for ventricular arrhythmia and RR = 0.51 for venous thromboembolism).

§ Although the average was 1.04, the confidence intervals for the contributing estimates were wide and evenly distributed about the null.

**Estimate, lower and upper bounds for the projected mortality difference (i.e. without denominator of total effect. Bounds were only estimated for medical events that appeared to explain the higher mortality for FGAs i.e. Relative Risk>1).

### Mortality

#### Epidemiology

Of the 20 included studies, 12 investigated mortality among antipsychotic users. Of these, 11 reported higher all-cause or non-cancer mortality for FGAs than SGAs, appearing as early as 30 days after antipsychotic initiation and lasting at least six months. In retrospective cohort studies of older community-dwelling adults, the hazard ratio was approximately 1.55 in the first 30 days after antipsychotic initiation, and ranged from 1.27 to 1.37 at six months [29], [34], [35] (Table 2). The relationship was more pronounced for doses above the median (HR = 1.67 to 1.73; median doses were unreported) and was attenuated for doses below the median (HR = 1.14 to 1.23). The corresponding absolute differences in six-month mortality varied from 7.3% (a statewide study in the mid-Atlantic U.S. [34]) to 2.5% (two province-wide studies in Canada [29], [35]), and a nationwide study of Australian Veterans Affairs beneficiaries reported a 10.6% difference in one year mortality [36]. While population differences in SGA-related mortality may explain these heterogeneous risk differences and relative risks [37], these studies consistently demonstrated a dose-dependent increase in mortality for FGAs early after initiation, lasting for at least six months in older community-dwelling adults.

Some analyses were restricted to dementia patients in the U.S. [34] or Canada [29], [35] and reported similar elevations in six-month mortality for FGAs compared to SGAs (HR = 1.23 to 1.29). One study of U.S. nursing home residents found elevations with FGAs only among patients with mostly mixed and vascular dementia [38], suggesting that the effect may be limited to a group of patients at elevated risk for cerebrovascular and cardiovascular disorders. Studies of U.S. veterans found higher mortality for haloperidol at 30 days [39] and 180 days [40], as compared to specific SGAs, while another study of U.S. veterans found no difference in mortality at one year follow up [41]. These results suggest that the excess mortality among dementia patients may be limited to the first six months after antipsychotic initiation.

Claims-based studies of antipsychotics have been criticized for their inability to adjust for important determinants of antipsychotic use that predict mortality in older adults—namely dementia severity, hallucinations, and delirium. Studies of U.S. nursing home residents also adjusted for measures of cognitive and functional decline as captured by federally mandated clinical assessments. Results from these studies [17], [38], [42], [43]—and others that further adjusted for facility-level characteristics [6], [17]—were similar to those conducted in community-based populations in terms of magnitude, timing, and dose-dependency [44]. A clinical study using validated measures of cognitive function and dementia symptoms found higher mortality with FGA use among nursing home residents, but not community-dwelling residents [45]. Psychoses, though, were found to strongly predict mortality in both samples. Confounding by delirium could be driven by the predominant use of haloperidol to treat agitation among intensive care unit patients with delirium [46], who have elevated six-month mortality rates of six-month mortality [47]. Hospital medication use is often not captured in claims data so some apparent new users may have in fact initiated haloperidol for delirium during an inpatient encounter. Because delirium is not well recorded in clinical or claims data, many of the studies reviewed here likely suffer from some degree of residual bias.

In sum, the evidence for elevated mortality in FGA users compared to SGA users is consistent across several national and clinical populations. Several strategies have been used to adjust for potential confounders and most of these results signal higher risk among FGA users, yet residual and unmeasured confounding cannot be completely excluded as alternate explanations. We now discuss the epidemiologic evidence for explaining this observed difference in mortality in terms of potential intermediate medical events.

### Stroke

#### Overview

From 2004 to 2012, 16 studies investigated the risk of stroke in FGA and SGA users, but only six met our inclusion criteria. Their results support higher stroke risk with FGAs than SGAs, but only within the first months after antipsychotic initiation (Table 2; HR = 1.03 to 1.91). The average incidence rate per 100 persons over six months among SGA users was 2.5, and among FGA users it was calculated to be 3.5 after applying the average relative risk for stroke (RR = 1.4). Assuming that the excess mortality for those who experience stroke after antipsychotic use is 17.8%, stroke may explain up to 6.7% of the mortality difference between FGAs and SGAs (Figure 2). The proportion mediated would be 7.4% if we expected poor sensitivity (Sn = 0.5) and a lower excess mortality of 10.0%; it would be 18.9% for the same sensitivity but a higher excess mortality of 26.0%.

#### Epidemiology

Six studies compared the risk for cerebrovascular events in populations with varying degrees of pre-existing cerebrovascular disease and duration of follow-up. Wang et al. followed low-income, community-dwelling older adults for 120 days after antipsychotic initiation [13] and reported a slightly higher risk of cerebrovascular events for FGAs than SGAs after 30 days (HR = 1.08; 95%CI 0.99 to 1.18) and through the end of follow-up (HR = 1.09; 95%CI 1.02 to 1.16). Finkel et al. followed Medicaid beneficiaries with dementia for three months and found higher risk for haloperidol compared to risperidone [10] (HR = 1.91; 95%CI 1.02 to 3.60). Another study followed older Medicaid beneficiaries for an average of three months and found higher risk of stroke with some FGAs as compared to SGAs; the risk was higher for phenothiazines (HR = 2.34; 95%CI 1.01 to 5.41) but not butyrophenones (HR = 1.44; 95%CI 0.55 to 3.76) [14]. Other studies followed patients for six months or longer and reported contrasting results. A study of nursing home residents found lower risk of stroke or transient ischemic attack for FGAs compared to SGAs (HR = 0.81; 95%CI 0.65 to 1.01) [18]. Gill et al. followed patients with dementia for an average of eight months and found no difference in risk [11] (HR = 0.99; 95%CI 0.79 to 1.23) even among high-risk subgroups defined by history of stroke or long-term care residence. A study based in Hong Kong followed dementia patients for an average of 2.4 years and also found no difference in risk [16] (HR = 0.93; 95%CI 0.52 to 1.67). These results may be explained by these studies' longer periods of follow-up, particularly if high-risk FGA patients experience stroke only during the first few months after antipsychotic initiation or if such patients were more likely to discontinue or switch their antipsychotic medication, which were both treated as censoring events in these analyses.

#### Biological plausibility

While the mechanism of antipsychotic-induced stroke is unclear, a recently proposed model postulated that antipsychotics could trigger cerebrovascular events in older adults with pre-existing medical comorbidities and concomitant medication use [21]. This is consistent with results from a recent case-case-time-control study of antipsychotic use and stroke in older adults [48], where cases essentially served as their own controls. FGAs, particularly haloperidol, the most widely used FGA, were associated with acute movement disorders that began soon after initiation. These extrapyramidal side-effects, as well as sedation by low-potency FGAs such as chlorpromazine, increase immobility, which can lead to or might exacerbate pre-existing cases of venous thromboembolism. Low-potency FGAs can also induce orthostatic hypotension, although it can occur with SGAs as well. Both of these complications can cause stroke, which is a leading cause of death.

#### Mortality associated with stroke

Based on the following data, we assumed that 20% of older adults who experience a stroke die within six months based on the following data: 80 to 90% of strokes are ischemic, and a study of patients hospitalized for ischemic stroke estimated their mortality as 7.4% within 30 days, 11.4% within 90 days, and 19.1% within one year. The corresponding proportions for hemorrhagic stroke were 18.8%, 24.6% and 31.8% [49]. Very little data on sudden death in ischemic stroke exists [50], but in the case of hemorrhagic stroke, 12% die before admission.

### Ventricular arrhythmia

#### Overview

Six studies investigated the risk for sudden cardiac death in FGA and SGA users. Of these, two met our inclusion criteria. These studies suggest that the excess risk for ventricular arrhythmias for FGAs compared to SGAs is limited to older, sicker populations during the first weeks of follow-up (Table 2; HR = 1.20). Similarly, FGAs and SGAs appear to increase the risk for ventricular arrhythmia in younger, healthier community dwelling patients followed for longer periods. The average incidence rate per 100 persons over six months among SGA users was 0.24, and among FGA users it was calculated to be 0.26 after applying the average relative risk for ventricular arrhythmia (RR = 1.1). Assuming that the excess mortality for those who experience ventricular arrhythmia after antipsychotic use is 87.8%, ventricular arrhythmia may explain up to 0.9% of the mortality difference between FGAs and SGAs (Figure 2). The proportion mediated would be 3.9% if we expected very poor sensitivity (Sn = 0.2) and a lower excess mortality of 81%; it would be 4.8% for the same sensitivity but a much higher excess mortality of 99.6%.

#### Epidemiology

The risk of out-of-hospital sudden cardiac death was investigated by Ray et al. in a retrospective cohort of non-psychotic, low-income persons with no history of prior cardiovascular disease [15]. This younger cohort (mean age 45.7) was followed for an average of two years and the results showed similar dose-dependent elevations in risk for current use of both FGAs (HR = 1.74; 95%CI 1.14 to 2.67) and SGAs (HR = 1.86; 95%CI 1.35 to 2.57) compared to non-use. Wang et al. investigated the risk of hospitalization for ventricular arrhythmia in a cohort of older Medicare beneficiaries and found higher risk for FGAs than SGAs but only within the first 30 days of follow up [13] (HR = 1.20; 95%CI 1.03 to 1.39). By 120 days of follow up, the effect had decreased and was no longer statistically significant (HR = 1.06; 95%CI 0.96 to 1.17).

#### Biological plausibility

FGAs such as thioridazine, pimozide, haloperidol, and droperidol are known for their dose-dependent risk of QT prolongation through blockage of potassium ion channels in cardiac tissue; ziprasidone is the only SGA associated with QT prolongation. In rare cases, QT prolongation above 450 msec increases the likelihood for a polymorphic ventricular arrhythmia known as Torsades de Pointes (TdP) [51], which commonly leads to ventricular arrhythmia and sudden cardiac death. The risk is higher among older patients with pre-existing cardiovascular disease [52].

#### Mortality associated with ventricular arrhythmia

Based on the following data, we assumed that 90% of older adults who experience a ventricular arrhythmia die within six months based on the following data: 80% percent of cardiac arrest cases occur in the community, where chances for survival are extremely low; one study estimated an overall survival of 6% [53]; for inpatients, the survival ranges from 5 to 35% [54].

### Venous thromboembolism

#### Overview

From 2005 to 2012, seven studies investigated the risk of venous thromboembolism (VTE) or pulmonary embolism (PE) in FGAs and SGAs. Of these, one met our inclusion criteria and reported higher VTE/PE risk for SGAs in the first six months after antipsychotic initiation (Table 2; HR = 0.5). The average incidence rate per 100 persons over six months among SGA users was 0.62, and among FGA users it was calculated to be 0.31, given this reported relative risk. Assuming that the excess mortality for those who experience VTE/PE after antipsychotic use is 13.8%, then the amount of the mortality difference between FGAs and SGAs explained by VTE/PE may be as high as −2.2%. Thus, VTE/PE does not appear to explain any of the higher mortality among FGAs as compared to SGAs (Figure 2).

#### Epidemiology

A retrospective cohort study of older nursing home residents assessed VTE risk over six months of follow-up after antipsychotic initiation [12]. Compared to non-users, SGAs showed nearly double the VTE risk in non-users (HR = 2.01; 95%CI 1.50 to 2.70) while FGAs showed no difference (HR = 1.02; 95%CI 0.67 to 1.55), even among low- and high-risk subgroups. While residual confounding cannot be ruled out as a possible explanation, these subgroup analyses suggest that strong confounding by disease severity or indication are unlikely.

#### Biological plausibility

The mechanism for antipsychotic-induced VTE—in particular why the risk may differ between FGAs and SGAs—is not known, but it may involve sedation and immobility in low-potency FGA users, increased weight gain and its sequale in SGA users, or other risk factors such as altered anticardiolipin antibody levels, fibrinolytic activity, or platelet aggregation [55]. VTE often begins as a deep vein thrombosis (DVT), which can be lethal when it leads to PE. Among older adults, PE occurs in a sizable number of VTE cases in both hospital and community populations [56].

#### Mortality associated with venous thromboembolism

Based on the following data, we assumed that 15% of older adults who experience a VTE die within six months based on the following data: a study of patients hospitalized for their first VTE estimated the case mortality as 3.6% by 30 days and 12.6% by one year among those without cancer [57]. Nearly one-quarter of those who develop PE present with sudden death and another quarter die within a year after discharge [58]. In the study reporting on VTE risk among antipsychotic users, 78% were diagnosed with DVT and 22% with PE.

### Myocardial infarction

#### Overview

We found five articles published from 2006 to 2012 that investigated myocardial infarction (MI) risk for FGAs and SGAs, two of which met our inclusion criteria. These studies are consistent with higher risk for FGAs than SGAs (Table 2; HR = 1.16 to 1.23). The average incidence rate per 100 persons over six months among SGA users was 1.0, and among FGA users it was calculated to be 1.2 after applying the average relative risk for MI (RR = 1.2). Assuming that the excess mortality for those who experience MI after antipsychotic use is 42.8%, then the proportion of the mortality difference between FGAs and SGAs explained by MI may be as high as 3.5% (Figure 2). The proportion mediated would be 4.2% if we expected poor sensitivity (Sn = 0.5) and a much lower excess mortality of 26.0%; it would be 9.5% for the same sensitivity but a much higher excess mortality of 59.6%.

#### Epidemiology

Two cohort studies have evaluated the risk of MI in both FGAs and SGAs. The Wang et al. study described earlier found a higher risk for FGA users at 120 days after antipsychotic initiation (HR = 1.16; 95%CI 0.91 to 1.48), but no difference in risk at 30 or 60 days [13]. The Huybrechts et al. study of nursing home residents also assessed the risk of hospitalization for MI at six months after initiation and found higher risk for FGAs compared to SGAs (HR = 1.23; 95%CI 0.82 to 1.82) [18]. This was the only study that reported an absolute rate of MI [18], which was low (2.0 cases per 100 person-years), so there may have been insufficient power to provide a more precise estimate.

#### Biological plausibility

The mechanisms by which antipsychotic use could lead to MI may involve effects on blood lipid levels, which may exacerbate atherosclerosis, or effects on platelets and other clotting factors leading to increased coagulability. MI is a leading cause of death.

#### Mortality associated with myocardial infarction

Based on the following data, we assumed that 45% of older adults who experience a myocardial infarction die within six months: in a study of older Medicare patients admitted for MI, 26.3% died within 30 days after discharge and an additional 20% died within the remaining year [5].

### Hip fracture

#### Overview

From 2004 to 2012, 12 studies investigated the association between falls or fracture risk in both FGA and SGA users, two of which met our inclusion criteria. These studies suggested that FGAs carry higher risk for hip fracture in nursing home residents soon after antipsychotic initiation (Table 2; HR = 1.27 to 1.61). Because falls and fractures occur more frequently among nursing home patients, this estimate may not apply to the general population. Nevertheless, the average incidence rate per 100 persons over six months among SGA users was 2.38, and among FGA users it was 3.33 after applying the average relative risk for hip fracture (RR = 1.4) (we averaged as-treated and intent-to-treat HR estimates reported in the same study; see File S2). Assuming that after antipsychotic use, the excess mortality for those who experience hip fracture is 17.8%, then hip fracture may explain up to 6.5% of the mortality difference between FGAs and SGAs (Figure 2). The proportion mediated would be 1.3% if we expected poor sensitivity (Sn = 0.5) and a much lower excess mortality of 3.1%; it would be 9.2% for the same sensitivity but a much higher excess mortality of 22.6%.

#### Epidemiology

Huybrechts et al. followed newly admitted nursing home residents in British Columbia for six months and found higher risk for FGAs than SGAs [17] (HR = 1.61; 95%CI 1.03 to 2.51), following patients until they experienced a hip fracture, died, discontinued their antipsychotic or started using an antipsychotic from the comparison group. When analyzed using the intention-to-treat approach, the hazard ratio was considerably lower (HR = 1.16; 95%CI 0.82 to 1.63). Another study followed nursing home residents without major psychotic disorders or cancer for six months and also found higher risk for FGAs (HR = 1.27; 95%CI 0.94 to 1.72) [18].

#### Biological plausibility

Acute extrapyramidal symptoms occur more frequently with FGAs [59]. Dystonia, parkinsonism, dyskinesia, and akathisia can manifest as early as the first few days after initiation or as late as three months. Such symptoms can cause gait disturbances and impair mobility and balance, which are risk factors for falls (and thus fractures) in older adults [60]. Fractures increase the risk for mortality in older adults.

#### Mortality associated with hip fracture

Based on the following data, we assumed that 20% of older adults who experience a hip fracture die within six months based on the following data: hip fracture increases mortality and 70% of the deaths occurring in the year following fracture happen within the first six months after injury [61]; at six months, the mortality among hip fracture cases ranges from 7.1% to 23%. Some of this mortality may be attributable to underlying comorbidity that increases risk for both hip fracture and mortality.

### Pneumonia

#### Overview

From 2004 to 2012, seven studies investigated the risk for pneumonia in both FGAs and SGAs. The three that met our inclusion criteria reported no differences in risk between FGAs and SGAs within the first six months after antipsychotic initiation. One study (Wang et al. 2007 [62]) reported lower risk in FGA users but only after 120 days follow-up, which could indicate that SGA users experience higher risk after three or four months following antipsychotic initiation but the average relative risk was null over follow-up. Taken together, these results suggest that pneumonia does not explain any of the higher mortality in FGAs as compared to SGAs (Figure 2).

#### Epidemiology

Three retrospective cohort studies evaluated the risk of pneumonia in FGA and SGA users. The Wang et al. study [62] of low-income older, frail, community-dwelling adults found no difference in risk at 30 (HR = 1.11; 95%CI 0.76 to 1.63) or 60 days (HR = 1.03; 95%CI 0.76 to 1.38). By 120 days the pneumonia risk was lower for FGAs as compared to SGAs (HR = 0.84; 95%CI 0.66 to 1.05). A study of newly admitted nursing home residents in British Columbia [17] showed no difference in risk at six months after antipsychotic initiation (HR = 1.03; 95%CI 0.62 to 1.69). Another study followed U.S. nursing home residents without cancer, schizophrenia, or bipolar disorder for up to six months and also found no difference in risk [18] (HR = 1.28; 95%CI 0.87 to 1.88).

#### Biological plausibility

The most plausible mechanism for possible differences in pneumonia risk for FGAs and SGAs is the higher frequency of extrapyramidal symptoms and sedation among FGA users. Extrapyramidal symptoms involving the pharyngeal musculature could lead to dysphagia, and excess sedation could lead to decreased cough-reflex, both of which are risk factors for aspiration pneumonia in older adults [63]. Another potential mechanism could involve altered cytokine profiles and immune response, but the supporting evidence for this pathway is incomplete as these studies have not comprehensively investigated how specific antipsychotics, FGAs or SGAs, influence inflammatory activity across in vivo, ex vivo, and in vitro systems [64].

#### Mortality associated with pneumonia

Based on the following data, we assumed that 20% of older adults who experience die within six months based on several lines of evidence. An observational study of nursing home residents with advanced dementia reported a 90-day mortality rate, after pneumonia diagnosis, ranging from 36% to 67% depending on the type of care received [65]; 51% of these cases had a do-not-hospitalize order. A review of community-acquired pneumonia reported a mortality rate of less than 1% for cases managed in outpatient settings [66]; among the 22 to 42% of patients in population-based studies with severe enough pneumonia to warrant inpatient or intensive care, mortality ranged from 14 to 54%. A large study of Medicare beneficiaries hospitalized for community-acquired pneumonia reported 11% mortality during hospitalization and 36% mortality over six-months among those discharged alive; at one year the total mortality was 41% [67]. Differences in pneumonia management and medical comorbidity, especially considering end-of-life decision making for older adults, may contribute to the wide variability in mortality among studies of inpatient pneumonia cases.

## Discussion

### Main findings

We found that older adults using FGAs were at higher risk for stroke, ventricular arrhythmia, myocardial infarction, and hip fracture as compared to SGAs. Using the lowest estimate of 2.5% for their overall difference in mortality, antipsychotic-induced stroke accounted for 6.7% of this difference, hip fracture for 6.5%, myocardial infarction for 3.5%, and ventricular arrhythmia for 0.9%. The lower and upper bounds that account for poor diagnostic sensitivity and other potential biases are 7.4% and 18.9% for stroke, 1.4% and 9.2% for hip fracture, 4.2% and 9.5% for myocardial infarction, and 3.9% and 4.8% for ventricular arrhythmia. Combined, these medical events explained about one-sixth (17%) of the mortality difference between FGAs and SGAs (assuming independent contributions), but this value could be as large as 42% given the limitations and uncertainties in the source data. Pneumonia and VTE did not account for any of the observed mortality difference. These results suggest that hip fracture, stroke, myocardial infarction, and ventricular arrhythmia are on the causal pathway to mortality, but that ventricular arrhythmia plays a minor role.

### Evidence gaps

Overall, our study was consistent with the findings of previous literature reviews of medical event risk in antipsychotic users [19], [21]–[25]. The following may explain why the medical events we reviewed did not explain more of the mortality difference: (1) the magnitude of the mortality difference may be biased from residual confounding through selective prescribing to FGA and SGA users based on socioeconomic status or prior health conditions (2) the difference in risk for each medical event may be similarly biased due to residual confounding; (3) most of the reviewed studies were performed in administrative data and used diagnostic billing records to assess the medical events, which typically results in low sensitivity and would underestimate the proportion mediated by a medical event [68]. In the absence of these potential biases, the data suggest that other medical events (e.g., bacterial infection, respiratory failure, renal failure, or neuroleptic malignant syndrome) may also contribute to the mortality difference.

While adequately powered randomized controlled studies evaluating medical event risk in both FGAs and SGAs would be ideal for characterizing the causal pathway to mortality, they are unlikely to be conducted and are possibly unethical in this setting. With the exception of stroke and pneumonia, we found few observational studies whose sample size, design, and analysis were suited to identify short-term drug effects (see inclusion criteria). Future studies with more robust methods would provide better evidence to characterize the risk of antipsychotic-related medical events and contribution to mortality.

The relative contributions by medical events to mortality most likely vary across populations with different indications, acuity, socioeconomic status, and quality of clinical care. While the association between antipsychotic type and mortality was characterized in several populations and subgroups, the studies of medical events were not. Among the included studies, stroke was the only event characterized in dementia patients. The other medical events were limited to either community-dwelling populations or nursing home residents.

### Limitations and strengths

This systematic review's results hinge on the quality of the included studies. Although each study reviewed adjusted for diagnoses, health service and medication use, it is possible that the individual studies suffered from residual or unmeasured confounding by risk factors for mortality or for the medical event studied. The algorithms used to classify medical comorbidity in administrative data, which were used by all of the included studies, often perform poorly (i.e. subject to misclassification) and will fail to completely control for confounding [69]. A study of community-dwelling Medicare beneficiaries showed that unmeasured confounding by BMI, smoking, and cognitive and functional impairment would underestimate the association between antipsychotic type and mortality [44]; the relationship between antipsychotic type and each of the medical events could be affected in similar ways. In some of the included studies [17], [29], [34], dementia, which is positively associated with increased cardiovascular risk and mortality, was more common in SGA users than FGA users (despite their differences in mean age). Other potential bias may come from risk factors for medical events, such as smoking, that independently predict mortality and are distributed differently between the general population and patients receiving antipsychotics. This sort of bias would distort our estimates of excess mortality but cannot be adequately controlled in this design. We have provided bounds and bias analyses to reflect these sources of uncertainty in our analysis.

The model used to estimate the proportion of the mortality difference mediated by medical events would be subject to any bias from unmeasured or residual confounding in the individual studies. Even in the absence of such bias, the model did not account for population heterogeneity between the studies reporting rates of medical events among antipsychotic users and the studies reporting mortality rates. In particular, the mortality rates were calculated from studies consisting of older adults from the general population; they may thus underestimate the expected mortality in antipsychotic users who did (or did not) experience a medical event. If this underestimation was more severe for those who did not experience a medical event, the proportion mediated would be inflated. Conversely, if this underestimation was more severe for those who experienced the medical event, the proportion mediated would be attenuated. If the mortality underestimation were similar in absolute terms for those with and without the medical event, the bias would cancel. The bounds and bias analyses reflect these sources of uncertainty.

Although it is clear from these analyses that ventricular arrhythmia plays a minor role, the bounds and bias analyses portray a sizeable amount of uncertainty for other medical events, especially in cases of extremely poor sensitivity of diagnostic algorithms used to extract diagnostic information from claims data (although for hip fracture this is less of a concern). Our most plausible estimates suggest that up to 42% of the smallest observed difference in mortality (2.5%) might be explained by these events. However, the unadjusted mortality difference is in fact larger in some populations (7.3%) and the corresponding upper bound would be 15% (the proportion mortality decreased because the projected mortality was held fixed while the overall difference in mortality was increased in this example). On the one hand, the large degree of unexplained mortality in either situation might demand investigation of other unsuspected adverse events. For instance, drug-drug interactions with antipsychotics may occur in the elderly whose age-related changes in drug metabolism and clearance may be exacerbated by complex medication regimens for comorbid conditions [70]. On the other hand, our inability to explain the mortality difference despite considering many known risks is also consistent with hypotheses of residual and unmeasured differences between patients who receive FGAs versus SGAs.

These limitations and uncertainty motivate a formal mediation analysis of individual-level data, with properly measured medical events, that would allow for confounding adjustment and would model the relationships between antipsychotic type, medical events, and mortality in the same dataset. Such an approach could also be performed for relevant population subgroups and confirmed across study populations. The current approach of synthesizing safety data could be extended to use more sophisticated methods such as Monte Caro simulation for estimating lower and upper bounds.

To our knowledge, our review is the first to quantify how important various medical events are in actually explaining mortality differences between FGAs and SGAs. Our results also demonstrate how the relationship between serious adverse drug events and mortality depend on their frequency, association with exposure, and lethality. The scope of prior reviews was limited to providing summary relative risks for the medical events and, in most cases, included studies whose designs were not optimally suited for assessing drug safety soon after initiation. We further improved upon these reviews by only summarizing studies that adjusted for measured confounders, employed a new user design, assessed covariates prior to antipsychotic initiation (or treatment change), and avoided selection bias and immortal person-time bias. These design choices protect against common threats to validity in observational studies and are recommended practice in pharmacoepidemiology [30], [71], [72].

This review focused on the mortality difference between FGAs and SGAs and, because these agents share mechanisms of action, the results may only partially apply to mortality differences between antipsychotic users versus non-users. To answer this pressing question, an alternate approach could focus on explaining the mortality difference between SGAs and placebo in clinical trials, and perhaps overcome their lack of power through pooling data at the individual or study level. However, this approach would face its own limitations: many of the placebo-controlled trials in dementia patients did not consistently report adverse events and their enrollment criteria often limit the relevance of their findings to patients seen in actual clinical practice [73]. The definitive effectiveness trial that compared individual SGAs for reducing behavioral and psychiatric symptoms in dementia lacked a placebo arm and was not designed to detect serious adverse events [74].

### Implications for clinical practice

We found consistently higher mortality for FGAs than SGAs in longitudinal claims-based studies. The few analyses that provided comparative data for medical events suggest that the mortality difference may be explained in part by stroke, hip fracture, acute myocardial infarction, and ventricular arrhythmia. These results emphasize the need for frequent monitoring of FGA users with pre-existing cardiovascular morbidity and functional decline, and that SGAs may represent safer initial options than FGAs after alternative therapies have been exhausted. As recommended by current treatment guidelines [2], patients started on either FGAs or SGAs should be evaluated early and repeatedly for whether a lower dose will suffice and whether continued use is warranted. This recommendation is buttressed by evidence that antipsychotic discontinuation in older adults reduces their mortality by as much as 32% in the following year [75].

## Conclusions

Our review summarized the literature on several serious adverse events in older antipsychotics and provided important contributions. First, newly published studies on antipsychotic-related mortality in nursing home residents that adjust for measures of functional and cognitive decline are consistent with previous findings. Second, despite the large body of research on antipsychotic-related medical events, very few studies followed recommended guidelines for reducing bias in observational studies of therapeutics. Last, and perhaps most important, we have shown that the published literature itself can be summarized to quantitatively compare potential *mechanisms* of drug-related mortality. Our results suggest the mortality difference between FGAs and SGAs may involve several pathways, and each influenced differently by the underlying medical event rate and the rate difference between FGAs and SGAs, as well as the excess mortality associated with the medical event. Much of the mortality difference between FGAs and SGAs was unexplained by the medical events included in this review. A formal mediation analysis using individual level data would overcome some of the limitations in our review.

## Supporting Information

Checklist S1 PRISMA checklist.
(PDF)

File S1 Pubmed search strategy.
(PDF)

File S2 Further details on synthesizing the evidence for the contribution of medical events to differences in mortality between FGA and SGA users.
(PDF)

File S3 Rationale for study eligibility criteria.
(PDF)

File S4 Proportion mediated model and source data.
(PDF)

File S5 Bias analysis: rationale and methods description.
(PDF)

File S6 Bias analysis: results and interpretation.
(PDF)

File S7 List of citations that received full-text review by event type and inclusion status.
(PDF)

File S8 Detailed descriptions of included studies.
(PDF)
