# Supplementary material for: Quantifying the Role of Adverse Events in the Mortality Difference between First and Second-Generation Antipsychotics in Older Adults: Systematic Review and Meta-Synthesis
Source: PLoS One. 2014 Aug 20;9(8):e105376. doi: 10.1371/journal.pone.0105376 (PMC4139353; doi:10.1371/journal.pone.0105376)
Supplement: Checklist S1 — PRISMA checklist. (PDF) [file pone.0105376.s001.pdf]

PRISMA 2009 Checklist

Section/topic	#	Checklist item	Section(s)
**TITLE**			
Title	1	Identify the report as a systematic review, meta-analysis, or both.	Title
**ABSTRACT**			
Structured summary	2	Provide a structured summary including, as applicable: background; objectives; data sources; study eligibility criteria, participants, and interventions; study appraisal and synthesis methods; results; limitations; conclusions and implications of key findings; systematic review registration number.	Abstract
**INTRODUCTION**			
Rationale	3	Describe the rationale for the review in the context of what is already known.	Introduction
Objectives	4	Provide an explicit statement of questions being addressed with reference to participants, interventions, comparisons, outcomes, and study design (PICOS).	Introduction
**METHODS**			
Protocol and registration	5	Indicate if a review protocol exists, if and where it can be accessed (e.g., Web address), and, if available, provide registration information including registration number.	NO
Eligibility criteria	6	Specify study characteristics (e.g., PICOS, length of follow-up) and report characteristics (e.g., years considered, language, publication status) used as criteria for eligibility, giving rationale.	Methods: Inclusion and exclusion criteria
Information sources	7	Describe all information sources (e.g., databases with dates of coverage, contact with study authors to identify additional studies) in the search and date last searched.	Methods: Search; Selection Process; Estimation of mortality rates for medical events
Search	8	Present full electronic search strategy for at least one database, including any limits used, such that it could be repeated.	Supporting Information S1
Study selection	9	State the process for selecting studies (i.e., screening, eligibility, included in systematic review, and, if applicable, included in the meta-analysis).	Methods: Selection Process; Estimation of mortality rates for medical events
Data collection process	10	Describe method of data extraction from reports (e.g., piloted forms, independently, in duplicate) and any processes for obtaining and confirming data from investigators.	Methods: Selection Process, Estimation of mortality rates for medical events

PRISMA 2009 Checklist

Section/topic	#	Checklist item	Section(s)
Data items	11	List and define all variables for which data were sought (e.g., PICOS, funding sources) and any assumptions and simplifications made.	Methods: Synthesizing the evidence for the contribution of medical events to differences in mortality between FGA and SGA users; Table 2; Supporting Information S4
Risk of bias in individual studies	12	Describe methods used for assessing risk of bias of individual studies (including specification of whether this was done at the study or outcome level), and how this information is to be used in any data synthesis.	Incorporated into inclusion criteria (see Methods: Inclusion and exclusion criteria, and Supporting Information S3); Supporting Information S5
Summary measures	13	State the principal summary measures (e.g., risk ratio, difference in means).	Methods: Synthesizing the evidence for the contribution of medical events to differences in mortality between FGA and SGA users
Synthesis of results	14	Describe the methods of handling data and combining results of studies, if done, including measures of consistency (e.g., I^2) for each meta-analysis.	Methods: Synthesizing the evidence for the contribution of medical events to differences in mortality between FGA and SGA users; Table 3; Supporting Information S2

PRISMA 2009 Checklist

Section/topic	#	Checklist item	Section(s)
Risk of bias across studies	15	Specify any assessment of risk of bias that may affect the cumulative evidence (e.g., publication bias, selective reporting within studies).	Discussion: Limitations and strengths; Table 1 shows biases present among excluded studies; Supporting information S6 discusses results of bias analysis
Additional analyses	16	Describe methods of additional analyses (e.g., sensitivity or subgroup analyses, meta-regression), if done, indicating which were pre-specified.	Methods: Bounds and bias analysis; Supporting information S5
**RESULTS**			
Study selection	17	Give numbers of studies screened, assessed for eligibility, and included in the review, with reasons for exclusions at each stage, ideally with a flow diagram.	Figure 1
Study characteristics	18	For each study, present characteristics for which data were extracted (e.g., study size, PICOS, follow-up period) and provide the citations.	Table 2, Supporting Information S8
Risk of bias within studies	19	Present data on risk of bias of each study and, if available, any outcome level assessment (see item 12).	Discussion: Limitations and strengths; Table 1 presents biases present among excluded studies. Supporting information S6 presents bias analysis results and interpretation
Results of individual studies	20	For all outcomes considered (benefits or harms), present, for each study: (a) simple summary data for each intervention group (b) effect estimates and confidence intervals, ideally with a forest plot.	Table 2; Table 3; Supporting Information; S6 Figure 3
Synthesis of results	21	Present results of each meta-analysis done, including confidence intervals and measures of consistency.	Table 3; Figure 2

PRISMA 2009 Checklist

Section/topic	#	Checklist item	Section(s)
Risk of bias across studies	22	Present results of any assessment of risk of bias across studies (see Item 15).	Discussion: Limitations and strengths; Table 1 presents biases present among excluded studies. Supporting information S6 presents bias analysis results and interpretation
Additional analysis	23	Give results of additional analyses, if done (e.g., sensitivity or subgroup analyses, meta-regression [see Item 16]).	Supporting Information S5 and S6 Tables 1 and 2, Figures 1-3
**DISCUSSION**			
Summary of evidence	24	Summarize the main findings including the strength of evidence for each main outcome; consider their relevance to key groups (e.g., healthcare providers, users, and policy makers).	Discussion: Main findings; Discussion: Implications for clinical practice
Limitations	25	Discuss limitations at study and outcome level (e.g., risk of bias), and at review-level (e.g., incomplete retrieval of identified research, reporting bias).	Discussion: Limitations and strengths
Conclusions	26	Provide a general interpretation of the results in the context of other evidence, and implications for future research.	Discussion: Evidence gaps; Discussion: Limitations and strengths; Conclusions
**FUNDING**			
Funding	27	Describe sources of funding for the systematic review and other support (e.g., supply of data); role of funders for the systematic review.	Included as part of manuscript submission process

From: Moher D, Liberati A, Tetzlaff J, Altman DG, The PRISMA Group (2009). Preferred Reporting Items for Systematic Reviews and Meta-Analyses: The PRISMA Statement. PLoS Med 6(6): e1000097. doi:10.1371/journal.pmed1000097. For more information, visit: **www.prisma-statement.org**.
