# Supplementary material for: Quantifying the Role of Adverse Events in the Mortality Difference between First and Second-Generation Antipsychotics in Older Adults: Systematic Review and Meta-Synthesis
Source: PLoS One. 2014 Aug 20;9(8):e105376. doi: 10.1371/journal.pone.0105376 (PMC4139353; doi:10.1371/journal.pone.0105376)
Supplement: File S1 — Pubmed search strategy. (PDF) [file pone.0105376.s002.pdf]

Supporting Information S1. Pubmed search strategy

(Executed October 09, 2012)

#1: Antipsychotic Terms

antipsychotics[MESH:exp] OR "Antipsychotic Agents" [Pharmacological Action] OR antipsychotic[TW] OR antipsychotics[TW] OR "anti-psychotic"[TW] OR "anti-psychotics"[TW] OR neuroleptic[TW] OR neuroleptics[TW] OR phenothiazine[TW] OR phenothiazines[TW] OR butyrophenone[TW] OR butyrophenones[TW] OR thioxanthene[TW] OR thioxanthenes[TW] OR dibenzoxazepine[TW] OR dibenzoxazepines[TW] OR acetophenazine[TW] OR chlorpromazine[TW] OR droperidol[TW] OR fluphenazine[TW] OR haloperidol[TW] OR loxapine[TW] OR mesoridazine[TW] OR molindone[TW] OR perphenazine[TW] OR pimozide[TW] OR thioridazine[TW] OR thiothixene[TW] OR trifluoperazine[TW] OR clozapine[TW] OR risperidone[TW] OR olanzapine[TW] OR quetiapine[TW] OR risperidone[TW] OR ziprasidone[TW] OR thorazine[TW] OR mellaril[TW] OR serentil[TW] OR stelazine[TW] OR prolixin[TW] OR permitil[TW] OR trilafon[TW] OR navane[TW] OR taractan[TW] OR loxitane[TW] OR haldol[TW] OR inapsine[TW] OR orap[TW] OR moban[TW] OR clozaril[TW] OR risperidal[TW] OR zyprexa[TW] OR seroquel[TW] OR geodon[TW] OR abilify[TW]

#2: Event Terms

fall[TW] OR falls[TW] OR fracture[TW] OR fractures[TW] OR "femoral fractures"[MESH:exp] OR pneumonia[TW] OR pneumonia[MESH:exp] OR "myocardial infarction"[TW] OR "mi"[TW] OR "heart attack"[TW] OR "myocardial infarction"[MESH:exp] OR "venous thromboembolism"[TW] OR "deep vein thrombosis"[TW] OR "phlebothrombosis"[TW] OR "pulmonary embolism"[TW] OR "vte" [TW] OR "dvt"[TW] OR "pe"[TW] OR "venous thromboembolism" [MESH:noexp] OR "pulmonary embolism"[MESH:noexp] OR "venous thrombosis"[MESH:noexp] OR "blood coagulation/drug effects" [MESH:noexp] OR "arrhythmia"[TW] OR "arrhythmias"[TW] OR "dysrhythmia"[TW] OR "dysrhythmias"[TW] OR "ventricular fibrillation"[TW] OR "ventricular tachycardia"[TW] OR "sudden death"[TW] OR "sudden cardiac death"[TW] OR "cardiac arrest"[TW] OR "torsades de pointes" OR "Arrhythmias, Cardiac"[MESH:exp] OR "heart arrest"[MESH:exp] OR "stroke"[TW] OR "cerebrovascular"[TW] OR "ischemic stroke"[TW] OR "ischaemic stroke"[TW] OR "subarachnoid hemorrhage"[TW] OR "intracerebral hemorrhage" [TW] OR "intracranial hemorrhage"[TW] OR "transient ischemic attack"[TW] OR "tia"[TW] OR "cve"[TW] OR "cva"[TW] OR cvae[TW] OR "cerebrovascular disorders"[MESH:exp] OR death[TW] or mortality[TW] OR survival[TW] OR "mortality"[MESH:exp] OR "fatal" [TW] OR "fatality"[TW]

#3: Risk Terms

risk[TW] OR incident[TW] OR incidence[TW] OR risk[MESH:exp] OR incidence[MESH:noexp]

#4: Epidemiologic Study Design Terms

"clinical trial"[TW] OR "clinical trials"[TW] OR "controlled trial"[TW] OR "controlled trials"[TW] OR rct[TW] OR rcts[TW] OR "intervention study"[TW] OR "intervention studies"[TW] OR

"observational study"[TW] OR "observational studies"[TW] OR registry[TW] OR registries[TW] OR register[TW] OR "population based"[TW] OR longitudinal[TW] OR longitudinally[TW] OR prospective[TW] OR prospectively[TW] OR retrospective[TW] OR follow[TW] OR followed[TW] OR "follow up" OR follow[TW] OR cohort[TW] OR "case control"[TW] OR "case cohort"[TW] OR "case only"[TW] OR "case series"[TW] OR "self controlled"[TW] OR "case crossover"[TW] OR "case time control"[TW] OR "case case time control"[TW] OR "clinical trials as topic"[MESH:exp] OR "intervention studies"[MESH:noexp]

#5: Evidence Synthesis Terms

"meta analysis"[TW] OR "systematic review"[TW] OR "Review Literature as Topic"[MESH:noexp]

#6: Final Search Strategy

(#1 AND #2 AND #3 AND (#4 OR #5)) AND 0000/00/00:2012/10/09[EDAT]
