# Supplementary material for: Quantifying the Role of Adverse Events in the Mortality Difference between First and Second-Generation Antipsychotics in Older Adults: Systematic Review and Meta-Synthesis
Source: PLoS One. 2014 Aug 20;9(8):e105376. doi: 10.1371/journal.pone.0105376 (PMC4139353; doi:10.1371/journal.pone.0105376)
Supplement: File S2 — Further details on synthesizing the evidence for the contribution of medical events to differences in mortality between FGA and SGA users. (PDF) [file pone.0105376.s003.pdf]

Supporting Information S2. Further details on synthesizing the evidence for the contribution of medical events to differences in mortality between FGA and SGA users

For studies reporting several relative risks stratified by length of follow-up, we selected the estimate whose corresponding follow-up use was closest to 180 days. For studies reporting estimates for use at baseline (e.g. approximating an intention-to-treat estimate) and until discontinuation (i.e. often referred to an as-treated analysis), we took an arithmetic average because the bias due to differences in discontinuation affects both estimates when predictors of discontinuation are not accounted for in comparative analyses. For studies reporting estimates stratified by structural taxonomy with no summary measure, we took an arithmetic average.
