# Supplementary material for: Quantifying the Role of Adverse Events in the Mortality Difference between First and Second-Generation Antipsychotics in Older Adults: Systematic Review and Meta-Synthesis
Source: PLoS One. 2014 Aug 20;9(8):e105376. doi: 10.1371/journal.pone.0105376 (PMC4139353; doi:10.1371/journal.pone.0105376)
Supplement: File S3 — Rationale for study eligibility criteria. (PDF) [file pone.0105376.s004.pdf]

Supporting Information S3. Rationale for study eligibility criteria

The randomized trial design is the widely accepted paradigm for causal inference. Short of experimentation, observational studies should be designed to reflect the randomized trial for the research question of interest. Recognizing that every research design and data source has limitations, we selected criteria that allow us to clearly interpret study findings in light of our research question: to what extent do various medical events mediate the causal pathway from antipsychotic type to all-cause mortality? Here, we clarify our rationale for inclusion criteria parts (4) through (6) as these were where most of the excluded articles fell short of inclusion.

Criterion 4. Studied "new users" of antipsychotic medications or required a washout-period of no use prior to cohort entry

The effect we want to explain is a short-term difference in risk that emerges during the first 6-months days after antipsychotic initiation, as observed in cohorts of antipsychotic users that had not initiated a prior antipsychotic within a defined window of time. Numerous studies showed that the mortality hazard declines with time. Epidemiologic methods[1] and empirical examples[2] show that including prevalent users—typically the ones who have tolerated a treatment well and show greater adherence—can yield biased results for understanding mortality and incident medical events that occur soon after the start of treatment. For studies that used a washout period to identify new users, we did not require a minimum window-length. In most cases this window was longer than 3 months.

Criterion 5. Adjusted for potential confounders that were assessed prior to antipsychotic initiation

Confounding in pharmacoepidemiology often arises because a risk factor for the outcome is also a determinant of whether or not patients receive treatment. An intermediate variable, however, is a variable that is affected by the treatment, which subsequently affects the outcome

itself. Epidemiologic practice has long cautioned against the control for intermediate variables of interest when the goal is to estimate a causal effect of exposure on outcome. The rationale for this recommendation is two-fold: 1) adjustment could block part of the effect of exposure that travels through the intermediate variable 2) adjustment could induce a selection bias[1] if predictors of that intermediate that also predict the outcome were not adjusted for[4], even in situations where the intermediate is not causally related to the outcome. When research questions do involve intermediates (e.g. mediation), special methods are required to avoid this latter type of bias[4]. Some cohort studies and many case-control studies we reviewed assessed exposure during the same time window as they assessed their covariates. Thus, it is likely that 50% of the time the covariates being adjusted were actually intermediate variables or occur after antipsychotic initiation. Not only does this increase the risk for bias due as described earlier, it also means that the adjustment has done little to adjust for variables that precede antipsychotic initiation and simultaneously predict mortality and the type of antipsychotic initiated at the start of follow-up (i.e. failed to adjust for confounding).

Criterion 6. Did not require a minimum period of survival after antipsychotic initiation.

Immortal person-time bias occurs when survival is built into an exposure definition and can lead to severe bias[5]. For example, consider a study that defines new users of antipsychotics as those who have contributed person-time for 1 year of follow-up, where antipsychotic use can occur at any time during that year. This is problematic for comparative designs investigating mortality as an outcome, because FGA and SGA users may differ in terms of *when* they actually initiate treatment or meet eligibility criteria for study inclusion. FGA and SGA users defined in this way may have differential mortality risk because of how they were selected into the study, even in the absence of confounding by pre-existing risk factors. Selection-bias can also occur when cohort entry requires survival for some fixed-time period past antipsychotic initiation. Such studies represent “survivor” cohorts where susceptible individuals in one exposure group could

be depleted during the fixed-time period prior to the start of follow-up. The effect observed after the start of follow up in such a study would not correspond to the effect in initiators of treatment.

References

1. Hernán, M. a., Hernández-Díaz, S., & Robins, J. M. (2004). A Structural Approach to Selection Bias. Epidemiology, 15(5), 615–625.

2. Johnson, E. S., Bartman, B. A., Briesacher, B. A., Fleming, N. S., Gerhard, T., Kornegay, C. J., Nourjah, P., et al. (2012). The incident user design in comparative effectiveness research. Pharmacoepidemiology and drug safety.

3. Hernán, M. A., Alonso, A., Logan, R., Grodstein, F., Michels, K. B., Willett, W. C., Manson, J. E., et al. (2008). Observational studies analyzed like randomized experiments: an application to postmenopausal hormone therapy and coronary heart disease. Epidemiology; 19(6), 766–79.

4. Vanderweele, T. J., & Vansteelandt, S. (2009). Conceptual issues concerning mediation, interventions and composition. Statistics and Its Interface, 2, 457–468.

5. Suissa, S. (2007). Immortal time bias in observational studies of drug effects. Pharmacoepidemiology and drug safety, 16(3), 241–9.
