# Supplementary material for: Quantifying the Role of Adverse Events in the Mortality Difference between First and Second-Generation Antipsychotics in Older Adults: Systematic Review and Meta-Synthesis
Source: PLoS One. 2014 Aug 20;9(8):e105376. doi: 10.1371/journal.pone.0105376 (PMC4139353; doi:10.1371/journal.pone.0105376)
Supplement: File S4 — Proportion mediated model and source data. (PDF) [file pone.0105376.s005.pdf]

Supporting Information S4. Proportion mediated model and source data

Proportion mediated = $\sum_m$ P[Y=1|M=m] × {P[M=m|A=1] - P[M=m|A=0]} ÷ {P[Y=1|M=1] - P[Y=1|M=0]}

Table 1. Description and source data for components used to estimate the proportion mediated

Notation	Description	Data and population used to estimate
A	Exposure: type of antipsychotic (0=SGA, 1=FGA)	---
M	Mediator: medical event occurrence within 6 months after initiating antipsychotic therapy (0=does not occur, 1=occurs)	---
Y	Outcome: vital status at 6 months after initiating antipsychotic therapy (0=alive, 1=deceased)	---
P[M=0|A=0]	6-month medical event free survival among SGA users	Studies based in retrospective claims data; SGA initiators with mean age over age 65
P[M=1|A=0]	6-month medical event risk among SGA users	Studies based in retrospective claims data; SGA initiators with mean age over age 65
P[M=0|A=1]	6-month medical event free survival among FGA users	Studies based in retrospective claims data; FGA initiators with mean age over age 65
P[M=1|A=1]	6-month medical event risk among FGA users	Studies based in retrospective claims data; FGA initiators with mean age over age 65
P[Y=1|M=0]	6-month mortality for persons who do not develop the medical event	U.S. Census mortality estimates; adults over age 65
P[Y=1|M=1]	6-month mortality for persons who develop the medical event	Clinical samples and general population studies; adults over 65 where possible
P[Y=1|A=0]	6-month mortality among SGA users	Studies based in retrospective claims data; SGA initiators with mean age over age 65
P[Y=1|A=1]	6-month mortality among FGA users	Studies based in retrospective claims data; FGA initiators with mean age over age 65
