# Supplementary material for: Quantifying the Role of Adverse Events in the Mortality Difference between First and Second-Generation Antipsychotics in Older Adults: Systematic Review and Meta-Synthesis
Source: PLoS One. 2014 Aug 20;9(8):e105376. doi: 10.1371/journal.pone.0105376 (PMC4139353; doi:10.1371/journal.pone.0105376)
Supplement: File S5 — Bias analysis: rationale and methods description. (PDF) [file pone.0105376.s006.pdf]

Supporting Information S5. Bias analysis: rationale and methods description

Rationale

The proportion of the mortality difference between first generation antipsychotic (FGA) users and second generation antipsychotic (SGA) users mediated by medical events was estimated by synthesizing estimates from two types of data sources. Type 1 data were collected through systematic review and consist of published studies describing the relationship between type of antipsychotic (A) and particular medical events (M), from which we extracted data on the risk of the medical event among SGA users ($Risk_{AM}$) and the relative risk for the medical event comparing FGA to SGA users (RR_{AM}); these estimates can be used to estimate the excess risk for medical events comparing FGA to SGA users (RD_{AM}). Other studies using these data sources reported on the overall difference in mortality comparing FGA to SGA users (RD_{AY}) which is used as the denominator for the proportion mediated.

Type 2 data came from published studies reporting data on the mortality rate among older adults who experienced these medical events; the mortality among older adults not experiencing a particular event was estimated by age-standardized annual mortality estimates (U.S. Census) for adults over age 65. These were then combined with type 1 data to estimate the proportion of six-month mortality mediated by each medical event, comparing those initiating FGAs to those initiating SGAs.

Studies contributing type 1 data were all performed using claims data which are frequently used for post-marketing safety studies because their large sample size facilitates the detection of rare adverse events. Because they are based on administrative data which can have poor diagnostic accuracy, these type 1 studies usually rely on algorithms with low-false positive classification rates (i.e. high specificity) that adequately predict diagnosis (i.e. high positive predictive value) but perform poorly in detecting cases (i.e. low sensitivity); these algorithms will typically

underestimate rates of medical events. These administrative data sources often do not contain information on important risk factors, making estimated relative risks vulnerable to unmeasured confounding. These limitations may introduce biases in the published studies which would carry over to analyses that synthesize their results.

Studies contributing type 2 data were usually from studies performed in clinical samples, health systems, or national register data (or published systematic reviews of these studies). None of the underlying populations in these studies were restricted to patients with dementia, or even older adults receiving antipsychotic therapy. Furthermore, the final estimates were based on interpolations from this data. As a result, the estimates of excess mortality associated with each medical event might also contain bias.

The implication for the present study is that bias in (a) estimates of excess risk RD_{AM} for each medical event comparing FGA to SGA users and (b) estimates of excess mortality RD_{MY} associated with each medical event, might carry over into estimates for the proportion of the mortality difference between antipsychotic types mediated by the medical events under study. We therefore undertook a thorough bias analysis to see how the estimated proportion mediated would change in response to correcting for the potential biases described above. We estimated lower and upper bounds that incorporate reasonable assumptions for the direction and magnitude of bias present in the studies contributing type 1 data, and in our interpolations and extrapolations used to produce estimates from type 2 data. We extended these results with graphical plots depicting how the corrected proportion mediated varies in relation to specific components of bias in the source data (i.e. amount of bias in $Risk_{AM}$, RR_{AM}, RD_{AM}, RD_{MY}, and RD_{AY}; descriptions of these quantities are provided in Supporting Information 5 Table 1) given more extreme scenarios. These results are shown in Supporting Information S6 Figures 1 and 2.

Last, we have also carried out a confirmatory analysis for the estimation method itself. The estimation method in the main text averages across type 1 studies to obtain the components $Risk_{AM}$ and RR_{AM} used to estimate the proportion mediated. An alternative approach is to estimate the proportion mediated for each study and then take an average across these results. In this second approach, one must still impute when a particular study does not report a necessary component (e.g. $Risk_{AM}$, for example). Also, the total effect RD_{AY} for a particular study was not always reported along with the medical event data. However, looking across studies it is possible in many cases to align data from articles that report medical event data ($Risk_{AM}$ and RR_{AM}) and total effect data (RD_{MY}) that arose from the same or similar data sources and cohort definitions. If we ignore the within-study total effects, we can likewise obtain an upper bound for the proportion mediated by computing these estimates using the smallest observed total effect as done in the main text. We provide a plot showing the individual proportion mediated for each study (and their mean) using the within-study total effect and the minimum and average total effect across all studies. Missing data for a given model component was imputed as the mean of the remaining studies contributing data for that medical event. These results are presented as graphical plots (see Supporting Information S6, Figure 3).

Because the goal of the study was to explain the higher mortality with FGA use, these analyses were only performed for medical events that appeared to have a higher risk with FGA use than SGA use (i.e. all except for pneumonia and venous thromboembolism).

Methods

Definitions

Table 1. Variables, paramaters, and other quantities used in bias analyses

Abbreviation	Notation	Description
A	---	Exposure: type of antipsychotic (0=SGA, 1=FGA)
M	---	Medical event: occurrence within 6 months after initiating antipsychotic therapy (0=does not occur, 1=occurs)
Y	---	Outcome: vital status at 6 months after initiating antipsychotic therapy (0=alive, 1=deceased)
RD_{AY}	P[Y=1|A=1] - P[Y=1|A=0]	Mortality difference comparing FGAs to SGA users
$Risk_{AM}$	P[M=1|A=0]	Risk of the medical event among SGA users
RR_{AM}	P[M=1|A=1] ÷ P[M=1|A=0]	Relative risk for medical event comparing FGA to SGA users
RD_{AM}	P[M=1|A=1] - P[M=1|A=0]	Risk difference for medical event comparing FGA to SGA users (i.e. excess risk for medical event)
RD_{MY}	P[Y=1|M=1] - P[Y=1|M=0]	Risk difference for mortality comparing FGA to SGA users (i.e. excess mortality)
PM	$\sum_m$ P[Y=1|M=m] × {P[M=m|A=1] - P[M=m|A=0]} ÷ RD_{AY}. Equivalently: RD_{AM} × RD_{MY} ÷ RD_{AY}	Proportion of the mortality difference comparing FGAs to SGA users medicated by a medical event (i.e. proportion mediated)
$Bias_{AM}$	RD_{AM}(observed) - RD_{AM}(true)	Amount of bias in RD_{AM} on the absolute scale
$Bias_{MY}$	RD_{MY}(observed) – RD_{MY}(true)	Amount of bias in RD_{MY} on the absolute scale

Lower and Upper Bounds

There was variability in the reported risk for the medical event and the relative risk comparing first- and second-generation agent users. The estimates of excess mortality associated with each medical event were based on extrapolations from different sources of data—census estimates, literature reviews, and empirical studies. We sought to provide lower and upper bounds for the proportion mediated by each medical event to reflect (a) potential bias in the source data and (b) our uncertainty in extrapolating from such data.

We assumed that biases in our estimates reflect systematic error, and not random error, and thus we do not employ probability distributions to provide bounds. Instead, we adjusted the reported absolute risk ($Risk_{AM}$) based on assumed levels of misclassification. We accounted for insensitivity but not non-specificity because researchers typically employ highly specific and predictive algorithms to detect rare medical events in claims data. Each study used different algorithms and the true sensitivities for these algorithms are unknown. We assumed an arbitrary sensitivity of 0.9 for hip fracture because it requires hospitalization for treatment and any related mortality will typically occur thereafter, so this event should be well captured in claims data. Stroke and myocardial infarction cases sometimes result in death before the individual reaches the hospital, and incident events that result in hospital care are sometimes difficult to distinguish from healthcare encounters for pre-existing conditions in administrative data, so we chose an arbitrary sensitivity of 0.5 for both events. Ventricular arrhythmia most often does result in sudden death, so we chose an arbitrary sensitivity of 0.2.

Because misclassification will not bias relative risks RR_{AM} for rare outcomes if very specific definitions are used, our concern was on the potential for potential unmeasured confounding of these estimates. The direction of potential unmeasured confounding in each individual study was unknown, so the average was retained as the best estimate when estimates from more

than one study were available. For consistency across mediators, we retained the single study estimate of RR_{AM} for ventricular arrhythmia. However, we note that correcting for positive confounding would decrease the estimated bounds, and correcting for negative confounding would increase them. To explore the contribution of values for RR_{AM} that are smaller and larger than the average value, bias analyses were undertaken and the results were displayed graphically (see next section, Bias Analysis Plots, for details).

For excess mortality (RD_{MY}), we estimated the lower bound as the 30 day mortality estimate for the medical event minus the one year mortality estimate for the general population, and the upper bound as the one year mortality estimate for the medical event minus the 30 day mortality for the general population (for myocardial infarction and ventricular arrhythmia there was not sufficient data to perform these calculations so we supplied our most reasonable estimates for the lower and upper estimates for excess mortality). Based on these input data, we estimated lower and upper bounds for the proportion mediated by each medical event (see Supporting Information S6 Table 1 for results). The overall mortality difference (RD_{AY}) comparing FGA to SGA users was fixed to the minimum reported value (.025) to provide logical upper bounds for these estimates. In Supporting Information S6 Table 2, we present these calculations with RD_{AY} also set to the maximum reported value (0.073) to portray how small the proportion mediated could be for populations with larger differences in mortality.

Bias Analysis Plots

In Supporting Information S6 Figure 1 we depict the hypothetical corrected or “true” proportion mediated that would be observed after adjusting for given amounts of bias (absolute scale) in RD_{AM} and RD_{MY}. These values were produced using the following expression: Corrected PM = $(RD_{AM} - Bias_{AM}) \times (RD_{MY} - Bias_{MY})$. The x-axis represents $Bias_{AM}$ ranging from bias consistent with a true RD_{AM} equal to zero (i.e. bias equal in magnitude but opposite sign to observed

value), to a true RD_{AM} of the same sign with magnitude of five times the observed value); these values more severe than those used to provide the upper and lower bounds for the corrected PM. The y-axis represents the hypothetical true PM in the bias scenario; and each line corresponds to a fixed value for $Bias_{MY}$ (ranging from -0.15 to 0.15, constrained such that the corrected RD_{MY} would lie between 0 and 1), which in most cases are equal to or less than the values used to provide the lower and upper bounds for the PM (the exception being myocardial infarction whose lower estimate for RD_{MY} is a few percentage points below the smallest plotted RD_{MY} scenario.

In Supporting Information S6 Figure 2 we depict the hypothetical corrected or “true” proportion mediated that would be observed after adjusting for given amounts of bias (absolute) in $Risk_{AM}$, RR_{AM}, and RD_{MY}. These values were produced using the following expression: Corrected PM = $((RR_{AM} \times Risk_{AM} - Risk_{AM}) - Bias_{AM}) \times (RD_{MY} - Bias_{MY})$. This approach decomposes RD_{AM} into RR_{AM} and $Risk_{AM}$, allowing us to separately consider the impacts from poor sensitivity of diagnostic algorithms and underestimating $Risk_{AM}$ and confounding of RR_{AM}. The x-axis represents bias in $Risk_{AM}$ ranging from its observed value to five times its observed value. The y-axis represents the true PM, and each line corresponds to a fixed value for RR_{AM} (three values were chosen: estimated RR_{AM}, equidistant on the log-scale between 1 and the estimated RR_{AM}, and between 2 and the estimated RR_{AM}). For each medical event, these plots were repeated for 3 fixed values of RD_{MY} corresponding to $Bias_{MY}$ values of -0.10, 0.00, and 0.10. A trellised plot was then produced where 3 columns represent fixed values of RD_{MY} and 4 rows represent the four medical events considered.

In both Figures, the estimated lower and upper bounds for the proportion mediated (as well as the estimated value from the observed data) were plotted as references to aid interpretation.
