# Supplementary material for: Quantifying the Role of Adverse Events in the Mortality Difference between First and Second-Generation Antipsychotics in Older Adults: Systematic Review and Meta-Synthesis
Source: PLoS One. 2014 Aug 20;9(8):e105376. doi: 10.1371/journal.pone.0105376 (PMC4139353; doi:10.1371/journal.pone.0105376)
Supplement: File S6 — Bias analysis: results and interpretation. (PDF) [file pone.0105376.s007.pdf]

Supporting Information S6. Bias analysis: results and interpretation

Results

Table 1. Lower and upper bounds for the proportion mediated by each medical event, with corresponding inputs used to quantify these bounds.

Mediator	Sensitivity in claims data	$Risk_{AM}$		Average RR_{AM}	Excess Mortality RD_{MY}			Proportion Mediated		
		Estimated	Adjusted		Estimated	Bounds Lower	Bounds Upper	Estimated	Bounds Lower	Bounds Upper
Hip Fracture	0.9	0.0306	0.0340	1.3	0.178	0.031	0.226	6.5%	1.3%	9.2%
Myocardial Infarction	0.5	0.0120	0.0240	1.2	0.428	0.260	0.596	3.4%	4.2%	9.5%
Stroke	0.5	0.0235	0.0460	1.4	0.178	0.100	0.260	6.7%	7.4%	18.9%
Ventricular Arrhythmia	0.2	0.0024	0.0120	1.1	0.878	0.810	0.996	0.8%	3.9%	4.8%
Total	---	---	---	---	---	---	---	17.4%	16.8%	42.4%

$Risk_{AM}$ is the absolute risk for the medical event among SGA users
RR_{AM} is the relative risk for the medical event comparing FGA to SGA users (reference)
RD_{MY} is the difference in mortality comparing those who experience a medical event to those who do not

Table 2. Upper and lower bounds for the proportion mediated by each medical event, with corresponding inputs used to quantify these bounds for total effect $Risk_{AY}$ at minimum and maximum reported values.

	Estimated PM		Lower Bound PM		Upper Bound PM	
	$Risk_{AY}$=.073	$Risk_{AY}$=.025	$Risk_{AY}$=.073	$Risk_{AY}$=.025	$Risk_{AY}$=.073	$Risk_{AY}$=.025
Hip Fracture	2.2%	6.5%	0.4%	1.3%	3.2%	9.2%
Myocardial Infarction	1.2%	3.5%	1.4%	4.2%	3.3%	9.5%
Stroke	2.3%	6.7%	2.5%	7.4%	6.5%	18.9%
Ventricular Arrhythmia	0.03%	0.8%	1.3%	3.9%	1.6%	4.8%
Total	6.0%	17.4	5.6%	16.8%	14.6%	42.4%

PM is the proportion mediated
RD_{AY} is the overall difference in mortality comparing FGA to SGA users (reference)

Figure 1. Proportion mediated corrected for bias in excess risk for mediator (RD_{AM}, FGA vs. SGA) and excess mortality for medical event (RD_{MY}). Corrected PM = (Observed RD_{AM} - $Bias_{RD\_AM}$) × (Observed RD_{MY} - $Bias_{RD\_MY}$). The dotted blue lines corresponds to the observed proportion mediated, the open blue circles mark the estimated lower and upper bounds, and the solid blue circle marks the scenario of no bias in RD_{AM} and RD_{MY}. Based on the sensitivities and relative risks used to estimate the lower and upper bounds, the most plausible values for $Bias_{RD\_AM}$ were -0.001 for hip fracture, -0.002 for myocardial infarction, - 0.009 for stroke, and - 0.001 for ventricular arrhythmia. The most plausible values for $Bias_{RD\_MY}$ used to estimate the lower and upper bounds for were, respectively, -0.04 and 0.14 for hip fracture, -0.17 and 0.17 for myocardial infarction, -0.08 and 0.08 for stroke, and -0.12 and 0.07 for ventricular arrhythmia.

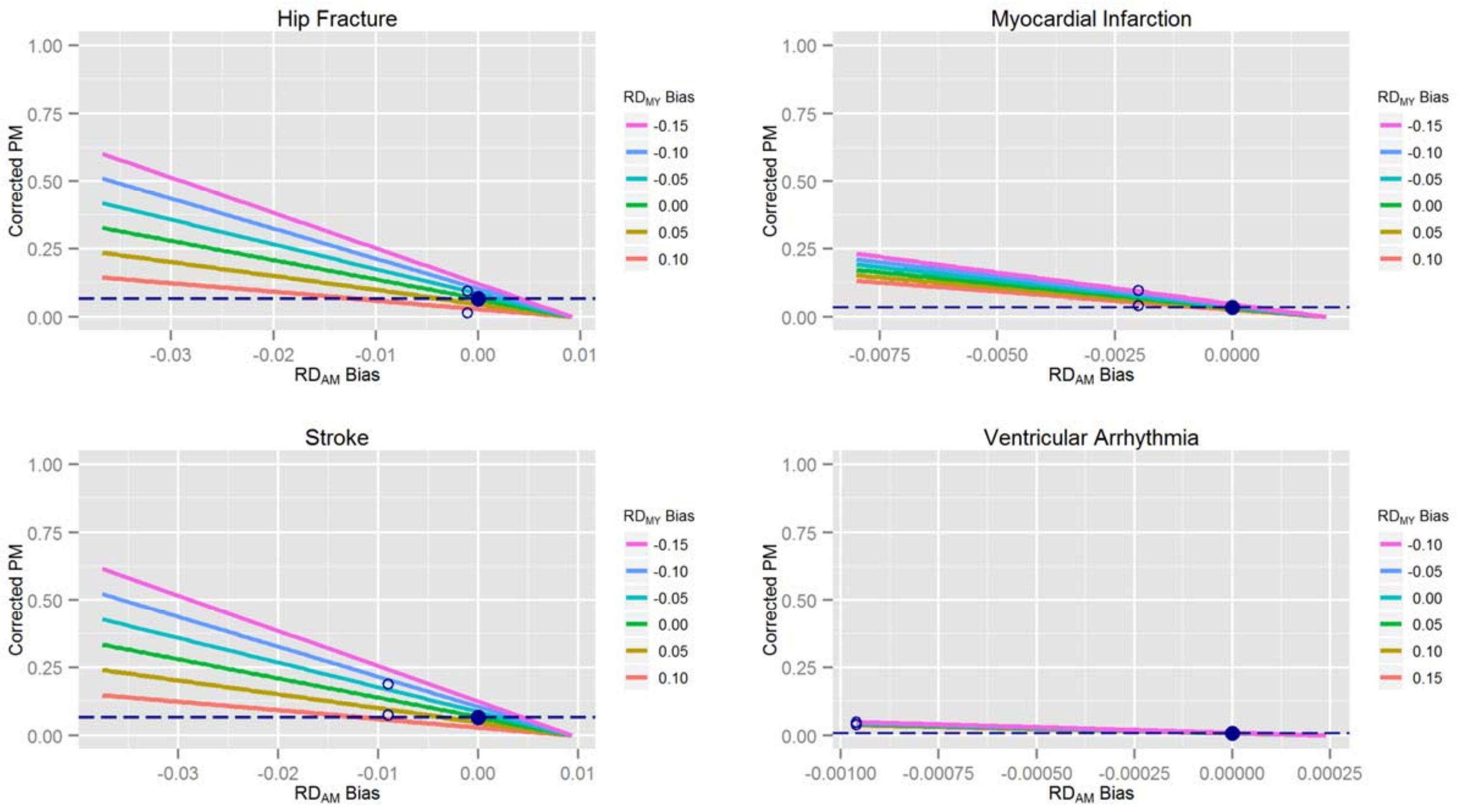

Figure 2. Proportion mediated as a function of risk of medical event among SGA users ($Risk_{AM}$), the relative risk for the mediator (RR_{AM}, FGA vs. SGA), and excess mortality for the medical event (RD_{MY}). PM = ($RR_{AM} \times Risk_{AM} - Risk_{AM}$) $\times RD_{MY}$. The dotted blue lines corresponds to the observed proportion mediated, the open blue circles mark the estimated upper and lower bounds, and the solid blue circle marks the scenario of no bias in RD_{AM} and RD_{MY}. Lines represent scenarios of fixed RR_{AM} at observed (green) and other values (hip fracture 1.14, 1.3, 1.61; myocardial infarction 1.1, 1.2, 1.55; stroke 1.18, 1.4, 1.67; ventricular arrhythmia 1.05, 1.1, 1.48).

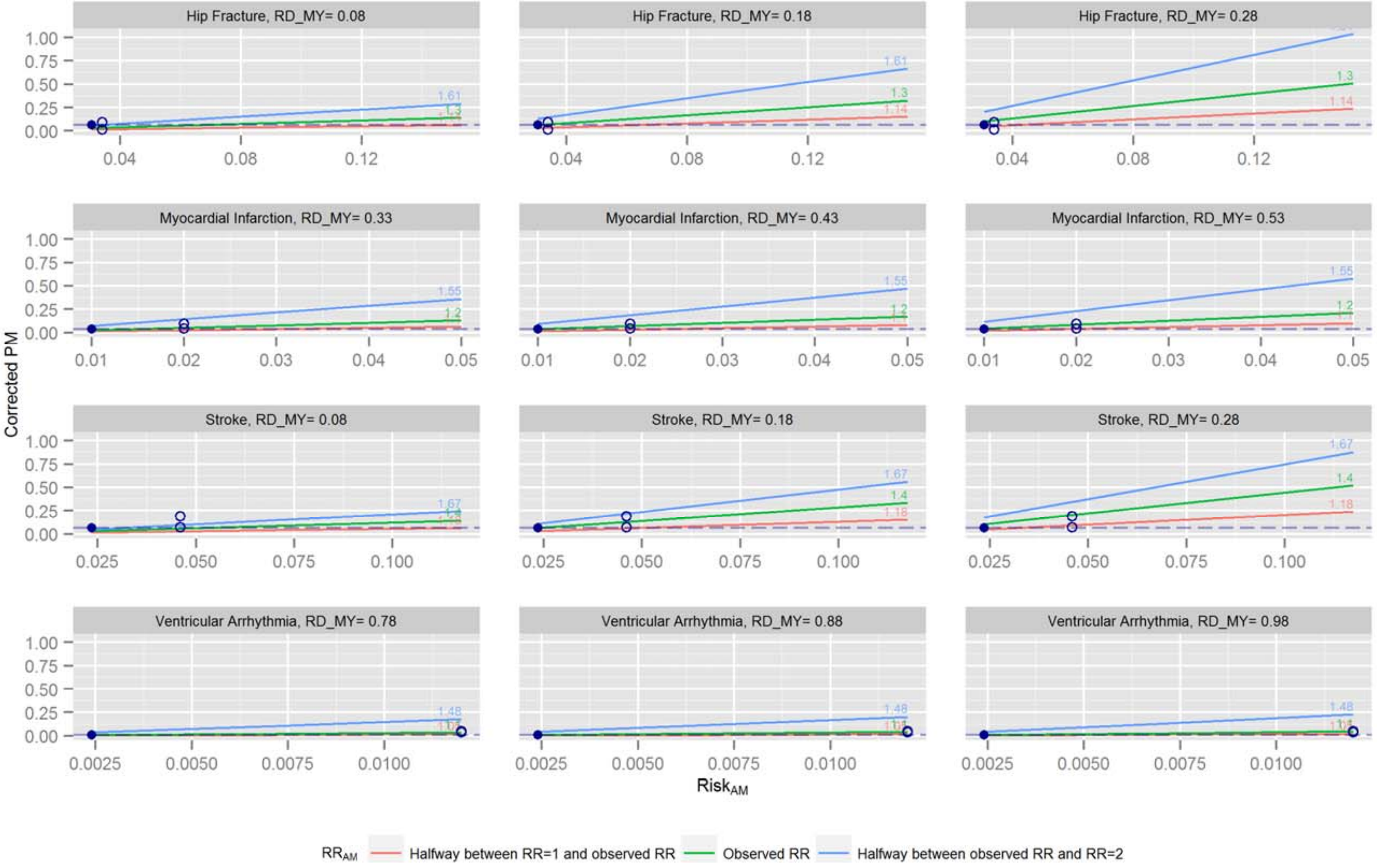

Figure 3. Proportion mediated using different estimation methods, applying the average total effect within studies examining the same mediator (top) and the lowest observed total effect of all studies .025 (bottom). The black dots represent the proportion mediated for individual studies, estimated from within-study data on risk. The black triangles represent the proportion mediated estimated as an average of these individual-study estimates. The blue triangles represent the proportion mediated estimated from the average values of study-level data on risk (as in the main text).

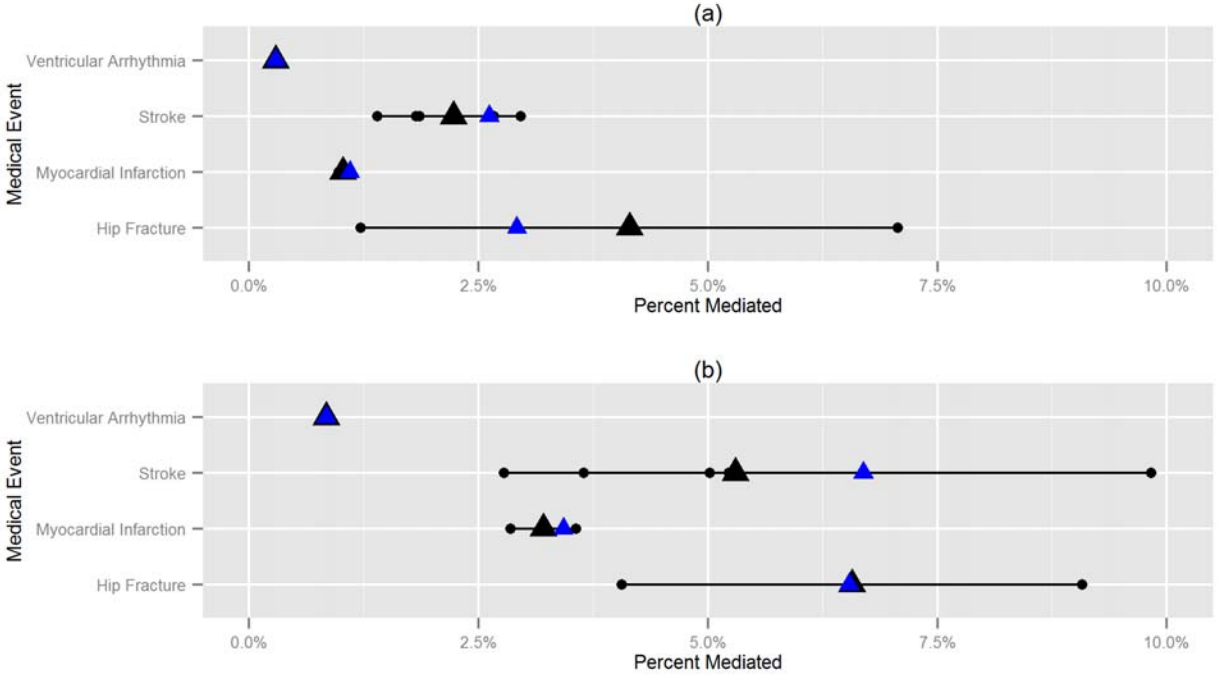

Interpretation

Hip Fracture

The observed proportion mediated by hip fracture was 6.5%, assuming the minimum observed total effect of 2.5%. Taking into account potential for misclassification of hip fracture (sensitivity=0.9) and inaccurate estimates of excess mortality due to hip fracture, the lower and upper bounds for the proportion mediated were, respectively, 1.3% and 9.2%. These bounds would decrease to 0.4% and 3.2% if all else was held fixed but the total effect was equal to 7.3%, the maximum value observed for the total effect. The bias analysis plots demonstrate that wider bounds are possible given more extreme scenarios such as lower sensitivity, or a negative bias in the relative risk for hip fracture. For example, consider the center panel of Supporting Information S6 Figure 2 where there is no bias in the excess mortality (RD_{MY}) due to hip fracture. Given a total effect of 2.5% and sensitivity of 0.5, if the relative risk for hip fracture

(RR_{AM}) were 1.61 instead of 1.3, the proportion mediated would be 15%. In this scenario there would be little net bias if RR_{MY} were biased upwards by 10% (left panel), but it would be much larger (21%) if RD_{MY} were biased downwards by 10% (right panel). If, in this latter scenario the sensitivity were 0.5 instead of our assumed value of 0.9, the proportion mediated would be 42%. Thus, while the bounds of 1.4% and 9.2% represent our best estimates for taking into account possible biases, the proportion mediated could be much higher in more extreme bias scenarios.

Myocardial Infarction

The observed proportion mediated by myocardial infarction was 3.5%, assuming the minimum observed total effect of 2.5%. Taking into account potential for misclassification of myocardial infarction (sensitivity=0.5) and inaccurate estimates of excess mortality due to myocardial infarction, the lower and upper bounds for the proportion mediated were, respectively, 4.2% and 9.5%. These bounds would decrease to 1.4% and 3.3% if all else was held fixed but the total effect was equal to 7.3%, the maximum value observed for the total effect. The bias analysis plots demonstrate that wider bounds are possible given more extreme scenarios such as lower sensitivity, or a negative bias in the relative risk for myocardial infarction. For example, consider the center panel of Supporting Information S6 Figure 2 where there is no bias in the excess mortality (RD_{MY}) due to myocardial infarction. Given a total effect of 2.5% and sensitivity of 0.5, if the relative risk for myocardial infarction (RR_{AM}) were 1.55 instead of 1.2, the proportion mediated would be 19%. In this scenario the proportion mediated would be 14% if RR_{MY} were also biased upwards by 10% (left panel); it would be 23% if RR_{MY} were biased downward by 10% (right panel). If in this latter scenario the sensitivity were 0.2 instead of our assumed value of 0.5, the proportion mediated would be 58%. Thus, while the bounds of 1.4% and 9.2% represent our best estimates for taking into account possible biases, the proportion mediated could be much higher in extreme bias scenarios.

Stroke

The observed proportion mediated by stroke was 6.7%, assuming the minimum observed total effect of 2.5%. Taking into account potential for misclassification of stroke (sensitivity=0.5) and inaccurate estimates of excess mortality due to stroke, the lower and upper bounds for the proportion mediated were, respectively, 7.4% and 18.9%. These bounds would decrease to 2.5% and 6.5% if all else was held fixed but the total effect was equal to 7.3%, the maximum value observed for the total effect. The bias analysis plots demonstrate that wider bounds are possible given more extreme scenarios such as lower sensitivity, or a negative bias in the relative risk for stroke. For example, consider the center panel of Supporting Information S6 Supporting Information S6 Figure 2 where there is no bias in the excess mortality (RD_{MY}) due to stroke. Given a total effect of 2.5% and sensitivity of 0.5, if the relative risk for stroke (RR_{AM}) were 1.67 instead of 1.4, the proportion mediated would be 22%. In this scenario the proportion mediated would be 10% if RR_{MY} were also biased upwards by 10% (left panel); it would be 35% if RR_{MY} were biased downward by 10% (right panel). If in this latter scenario the sensitivity were 0.2 instead of our assumed value of 0.5, the proportion mediated would be 89%. Thus, while the bounds of 7.4% and 18.9% represent our best estimates for taking into account possible biases, the proportion mediated could be much higher in extreme bias scenarios.

Ventricular Arrhythmia

The observed proportion mediated by ventricular arrhythmia was 0.9%, assuming the minimum observed total effect of 2.5%. Taking into account potential for misclassification of ventricular arrhythmia (sensitivity=0.2) and inaccurate estimates of excess mortality due to ventricular arrhythmia, the lower and upper bounds for the proportion mediated were, respectively, 3.9% and 4.8%. These bounds would decrease to 1.3% and 4.8% if all else was held fixed but the total effect was equal to 7.3%, the maximum value observed for the total effect. The bias analysis plots demonstrate that wider bounds are possible given extreme scenarios such as

lower sensitivity, or a negative bias in the relative risk for ventricular arrhythmia. For example, consider the center panel of Supporting Information S6 Figure 2 where there is no bias in the excess mortality (RD_{MY}) due to ventricular arrhythmia. Given a total effect of 2.5% and sensitivity of 0.2, if the relative risk for ventricular arrhythmia (RR_{AM}) were 1.48 instead of 1.05, the proportion mediated would be 20%. In this scenario the proportion mediated would be 18% if RR_{MY} were also biased upwards by 10% (left panel); it would be 22% if RR_{MY} were biased downward by 10% (right panel). Thus, while the bounds of 1.3% and 4.8% represent our best estimates for taking into account possible biases, the proportion mediated could be much higher in extreme bias scenarios.

Overall

We summed across the observed estimate for the proportion mediated for each medical event, and did the same for the lower bound and upper bounds. The observed estimate is that 17.4% of the total effect, the overall mortality difference between FGA and SGA uses is mediated by hip fracture, myocardial infarction, stroke, and ventricular arrhythmia. Accounting for potential biases in the source data suggest that this quantity may be as low as 16.8% or as high as 42.4% if the total effect to be explained was indeed 2.5%--the lowest reported in published studies. If the total effect itself were biased downwards (from the maximum value observed, 7.3%) then the bounds would be as low as 5.6% and 14.6%. Thus, it appears that our reported estimate, 17.4% is consistent with several different bias scenarios. Of course, if bias scenarios for each mediator simultaneously led to downwards bias given more extreme scenarios, then this total effect might be fully explained by these medical events. Similarly, if bias scenarios for each mediator simultaneously led towards upwards bias given more extreme scenarios, then little of this total effect might be explained by these medical events.

The bounds reported in Supporting Information S6 Tables 1 and 2 reflect the poor sensitivity of algorithms used to detect medical events, and potential for unmeasured confounding in studies that estimate relative risks of medical events comparing FGA and SGA users, as well as uncertainty in the extrapolation of mortality data to obtain estimates for excess mortality associated with each medical event. Given these, it does not appear that any single medical event can fully explain the mortality difference between FGAs and SGAs; rather their contributions appear quite small. This conclusion is most robust for ventricular arrhythmia, in particular, because only extreme bias scenarios would change these conclusions.

The bounds are widest for stroke, the medical event for which there is the most data available. This may reflect residual bias or effect modification among the individual studies, as well as heterogeneity in definitions used for stroke. Future studies for stroke and other medical events could yield useful data by harmonizing outcome measures, reporting stratum-specific estimates by age or other potential effect modifiers, and measuring and adjusting for important confounders such as smoking, delirium, and biological markers of risk (e.g. LDL, blood pressure, etc.).

We compared estimators that (a) use individual level study data to compute the proportion mediated within each study and then average across these results (b) average across individual level study data and compute the proportion mediated from these summary estimates. The two approaches gave similar results in terms of magnitude and ranking among the different medical events. As expected, the proportion mediated results were smaller for within-study total effects than for those using the overall lowest total effect.
