# Supplementary material for: Quantifying the Role of Adverse Events in the Mortality Difference between First and Second-Generation Antipsychotics in Older Adults: Systematic Review and Meta-Synthesis
Source: PLoS One. 2014 Aug 20;9(8):e105376. doi: 10.1371/journal.pone.0105376 (PMC4139353; doi:10.1371/journal.pone.0105376)
Supplement: File S7 — List of citations that received full-text review by event type and inclusion status. (PDF) [file pone.0105376.s008.pdf]

Supporting Information S7. List of citations that received full-text review by event type and inclusion status

Mortality - Included

1. Aparasu RR, Chatterjee S, Mehta S, Chen H. Risk of Death in Dual-eligible Nursing Home Residents Using Typical or Atypical Antipsychotic Agents. Medical care. 2012;50(11):961-9. Epub 2012/10/11. doi: 10.1097/MLR.0b013e31826ec185. PubMed PMID: 23047786.

2. Gill SS, Bronskill SE, Normand SL, Anderson GM, Sykora K, Lam K, et al. Antipsychotic drug use and mortality in older adults with dementia. Annals of internal medicine. 2007;146(11):775-86. Epub 2007/06/06. PubMed PMID: 17548409.

3. Hollis J, Grayson D, Forrester L, Brodaty H, Touyz S, Cumming R. Antipsychotic medication dispensing and risk of death in veterans and war widows 65 years and older. The American journal of geriatric psychiatry. 2007;15(11):932-41. Epub 2007/11/03. doi: 10.1097/JGP.0b013e31813547ca. PubMed PMID: 17974865.

4. Huybrechts KF, Brookhart MA, Rothman KJ, Silliman RA, Gerhard T, Crystal S, et al. Comparison of different approaches to confounding adjustment in a study on the association of antipsychotic medication with mortality in older nursing home patients. American journal of epidemiology. 2011;174(9):1089-99. Epub 2011/09/22. doi: 10.1093/aje/kwr213. PubMed PMID: 21934095; PubMed Central PMCID: PMCPMC3243935.

5. Huybrechts KF, Gerhard T, Crystal S, Olfson M, Avorn J, Levin R, et al. Differential risk of death in older residents in nursing homes prescribed specific antipsychotic drugs: population based cohort study. BMJ (Clinical research ed). 2012;344:e977. Epub 2012/03/01. doi: 10.1136/bmj.e977. PubMed PMID: 22362541; PubMed Central PMCID: PMCPMC3285717.

6. Huybrechts KF, Rothman KJ, Silliman RA, Brookhart MA, Schneeweiss S. Risk of death and hospital admission for medical events after initiation of psychotropic medications in older adults admitted to nursing homes. CMAJ. 2011;183(7):E411-9. Epub 2011/03/30. doi: 10.1503/cmaj.101406. PubMed PMID: 21444611; PubMed Central PMCID: PMCPMC3080558.

7. Kales HC, Kim HM, Zivin K, Valenstein M, Seyfried LS, Chiang C, et al. Risk of mortality among individual antipsychotics in patients with dementia. The American journal of psychiatry. 2012;169(1):71-9. Epub 2011/12/24. doi: 10.1176/appi.ajp.2011.11030347. PubMed PMID: 22193526.

8. Kales HC, Valenstein M, Kim HM, McCarthy JF, Ganoczy D, Cunningham F, et al. Mortality risk in patients with dementia treated with antipsychotics versus other psychiatric medications. The American journal of psychiatry. 2007;164(10):1568-76; quiz 623. Epub 2007/09/28. doi: 10.1176/appi.ajp.2007.06101710. PubMed PMID: 17898349.

9. Liperoti R, Onder G, Landi F, Lapane KL, Mor V, Bernabei R, et al. All-cause mortality associated with atypical and conventional antipsychotics among nursing home residents with dementia: a retrospective cohort study. The Journal of clinical psychiatry. 2009;70(10):1340-7. Epub 2009/11/13. doi: 10.4088/JCP.08m04597yel. PubMed PMID: 19906339.

10. Pratt N, Roughead EE, Ryan P, Salter A. Antipsychotics and the risk of death in the elderly: an instrumental variable analysis using two preference based instruments. Pharmacoepidemiol Drug Saf. 2010;19(7):699-707. Epub 2010/06/29. doi: 10.1002/pds.1942. PubMed PMID: 20583208.

11. Rossom RC, Rector TS, Lederle FA, Dysken MW. Are all commonly prescribed antipsychotics associated with greater mortality in elderly male veterans with dementia? Journal of the American Geriatrics Society. 2010;58(6):1027-34. Epub 2010/05/22. doi: 10.1111/j.1532-5415.2010.02873.x. PubMed PMID: 20487081.

12. Schneeweiss S, Setoguchi S, Brookhart A, Dormuth C, Wang PS. Risk of death associated with the use of conventional versus atypical antipsychotic drugs among elderly patients. CMAJ. 2007;176(5):627-32. Epub 2007/02/28. doi: 10.1503/cmaj.061250. PubMed PMID: 17325327; PubMed Central PMCID: PMCPMC1800321.

13. Wang PS, Schneeweiss S, Avorn J, Fischer MA, Mogun H, Solomon DH, et al. Risk of death in elderly users of conventional vs. atypical antipsychotic medications. The New England journal of medicine. 2005;353(22):2335-41. Epub 2005/12/02. doi: 10.1056/NEJMoa052827. PubMed PMID: 16319382.

Mortality - Excluded

1. Barnett MJ, Perry PJ, Alexander B, Kaboli PJ. Risk of mortality associated with antipsychotic and other neuropsychiatric drugs in pneumonia patients. J Clin Psychopharmacol. 2006;26(2):182-7. Epub 2006/04/25. doi: 10.1097/01.jcp.0000203598.43314.34. PubMed PMID: 16633149.

2. Hollis J, Forrester L, Brodaty H, Touyz S, Cumming R, Grayson D. Risk of death associated with antipsychotic drug dispensing in residential aged care facilities. The Australian and New Zealand journal of psychiatry. 2007;41(9):751-8. Epub 2007/08/10. doi: 10.1080/00048670701519864. PubMed PMID: 17687661.

3. Hollis J, Grayson D, Forrester L, Brodaty H, Touyz S, Cumming R. Antipsychotic medication dispensing and risk of death in veterans and war widows 65 years and older. The American journal of geriatric psychiatry : official journal of the American Association for Geriatric Psychiatry. 2007;15(11):932-41. Epub 2007/11/03. doi: 10.1097/JGP.0b013e31813547ca. PubMed PMID: 17974865.

4. Marras C, Gruneir A, Wang X, Fischer H, Gill SS, Herrmann N, et al. Antipsychotics and mortality in Parkinsonism. The American journal of geriatric psychiatry : official journal of the American Association for Geriatric Psychiatry. 2012;20(2):149-58. Epub 2012/01/26. doi: 10.1097/JGP.0b013e3182051bd6. PubMed PMID: 22273735.

5. Musicco M, Palmer K, Russo A, Caltagirone C, Adorni F, Pettenati C, et al. Association between prescription of conventional or atypical antipsychotic drugs and mortality in older persons with Alzheimer's disease. Dementia and geriatric cognitive disorders. 2011;31(3):218-24. Epub 2011/04/09. doi: 10.1159/000326213. PubMed PMID: 21474930.

6. Raivio MM, Laurila JV, Strandberg TE, Tilvis RS, Pitkala KH. Neither atypical nor conventional antipsychotics increase mortality or hospital admissions among elderly patients with dementia: a two-year prospective study. The American journal of geriatric

psychiatry : official journal of the American Association for Geriatric Psychiatry. 2007;15(5):416-24. Epub 2007/04/28. doi: 10.1097/JGP.0b013e31802d0b00. PubMed PMID: 17463191.

7. Simoni-Wastila L, Ryder PT, Qian J, Zuckerman IH, Shaffer T, Zhao L. Association of Antipsychotic Use With Hospital Events and Mortality Among Medicare Beneficiaries Residing in Long-Term Care Facilities. AmJGeriatrPsychiatry. 2009;17(5). doi: 10.1097/JGP.0b013e31819b8936. PubMed PMID: WOS:000267418900003.

8. Trifiro G, Verhamme KM, Ziere G, Caputi AP, Ch Stricker BH, Sturkenboom MC. All-cause mortality associated with atypical and typical antipsychotics in demented outpatients. Pharmacoepidemiol Drug Saf. 2007;16(5):538-44. Epub 2006/10/13. doi: 10.1002/pds.1334. PubMed PMID: 17036366.

Cerebrovascular – Included

1. Chan MC, Chong CS, Wu AY, Wong KC, Dunn EL, Tang OW, et al. Antipsychotics and risk of cerebrovascular events in treatment of behavioural and psychological symptoms of dementia in Hong Kong: a hospital-based, retrospective, cohort study. International journal of geriatric psychiatry. 2010;25(4):362-70. Epub 2009/08/04. doi: 10.1002/gps.2347. PubMed PMID: 19650162.

2. Finkel S, Kozma C, Long S, Greenspan A, Mahmoud R, Baser O, et al. Risperidone treatment in elderly patients with dementia: relative risk of cerebrovascular events versus other antipsychotics. International psychogeriatrics / IPA. 2005;17(4):617-29. Epub 2005/10/06. doi: 10.1017/s1041610205002280. PubMed PMID: 16202186.

3. Gill SS, Rochon PA, Herrmann N, Lee PE, Sykora K, Gunraj N, et al. Atypical antipsychotic drugs and risk of ischaemic stroke: population based retrospective cohort study. BMJ (Clinical research ed). 2005;330(7489):445. Epub 2005/01/26. doi: 10.1136/bmj.38330.470486.8F. PubMed PMID: 15668211; PubMed Central PMCID: PMCPMC549652.

4. Huybrechts KF, Schneeweiss S, Gerhard T, Olfson M, Avorn J, Levin R, et al. Comparative safety of antipsychotic medications in nursing home residents. Journal of the American Geriatrics Society. 2012;60(3):420-9. Epub 2012/02/15. doi: 10.1111/j.1532-5415.2011.03853.x. PubMed PMID: 22329464; PubMed Central PMCID: PMCPMC3302976.

5. Sacchetti E, Trifiro G, Caputi A, Turrina C, Spina E, Cricelli C, et al. Risk of stroke with typical and atypical anti-psychotics: a retrospective cohort study including unexposed subjects. Journal of psychopharmacology (Oxford, England). 2008;22(1):39-46. Epub 2008/01/12. doi: 10.1177/0269881107080792. PubMed PMID: 18187531.

6. Wang PS, Schneeweiss S, Setoguchi S, Patrick A, Avorn J, Mogun H, et al. Ventricular arrhythmias and cerebrovascular events in the elderly using conventional and atypical antipsychotic medications. J Clin Psychopharmacol. 2007;27(6). WOS:000251181600024.

Cerebrovascular – Excluded

1. Douglas IJ, Smeeth L. Exposure to antipsychotics and risk of stroke: self controlled case series study. BMJ (Clinical research ed). 2008;337:a1227. Epub 2008/08/30. doi: 10.1136/bmj.a1227. PubMed PMID: 18755769; PubMed Central PMCID: PMCPMC2526549.

2. Herrmann N, Mamdani M, Lanctot KL. Atypical antipsychotics and risk of cerebrovascular accidents. The American journal of psychiatry. 2004;161(6):1113-5. Epub 2004/06/01. PubMed PMID: 15169702.

3. Kleijer BC, van Marum RJ, Egberts AC, Jansen PA, Knol W, Heerdink ER. Risk of cerebrovascular events in elderly users of antipsychotics. Journal of psychopharmacology (Oxford, England). 2009;23(8):909-14. Epub 2008/07/19. doi: 10.1177/0269881108093583. PubMed PMID: 18635700.

4. Kolanowski A, Fick D, Waller JL, Ahern F. Outcomes of antipsychotic drug use in community-dwelling elders with dementia. Archives of psychiatric nursing. 2006;20(5):217-25. Epub 2006/10/03. doi: 10.1016/j.apnu.2006.04.004. PubMed PMID: 17010825.

5. Laredo L, Vargas E, Blasco AJ, Aguilar MD, Moreno A, Portoles A. Risk of cerebrovascular accident associated with use of antipsychotics: population-based case-control study. Journal of the American Geriatrics Society. 2011;59(7):1182-7. Epub 2011/07/02. doi: 10.1111/j.1532-5415.2011.03479.x. PubMed PMID: 21718267.

6. Liperoti R, Gambassi G, Lapane KL, Chiang C, Pedone C, Mor V, et al. Cerebrovascular events among elderly nursing home patients treated with conventional or atypical antipsychotics. The Journal of clinical psychiatry. 2005;66(9):1090-6. Epub 2005/09/29. PubMed PMID: 16187764.

7. Mehta S, Johnson ML, Chen H, Aparasu RR. Risk of cerebrovascular adverse events in older adults using antipsychotic agents: a propensity-matched retrospective cohort study. The Journal of clinical psychiatry. 2010;71(6):689-98. Epub 2010/06/25. doi: 10.4088/JCP.09m05817yel. PubMed PMID: 20573328.

8. Percudani M, Barbui C, Fortino I, Tansella M, Petrovich L. Second-generation antipsychotics and risk of cerebrovascular accidents in the elderly. J Clin Psychopharmacol. 2005;25(5):468-70. Epub 2005/09/15. PubMed PMID: 16160623.

9. Pratt NL, Roughead EE, Ramsay E, Salter A, Ryan P. Risk of hospitalization for stroke associated with antipsychotic use in the elderly: a self-controlled case series. Drugs & aging. 2010;27(11):885-93. Epub 2010/10/23. doi: 10.2165/11584490-000000000-00000. PubMed PMID: 20964462.

10. Wu CS, Wang SC, Gau SS, Tsai HJ, Cheng YC. Association of Stroke with the Receptor-Binding Profiles of Antipsychotics-A Case-Crossover Study. Biol Psychiatry. 2012. Epub 2012/08/11. doi: 10.1016/j.biopsych.2012.07.006. PubMed PMID: 22877922.

Ventricular Arrhythmia / Sudden Cardiac Death - Included

1. Ray WA, Chung CP, Murray KT, Hall K, Stein CM. Atypical antipsychotic drugs and the risk of sudden cardiac death. The New England journal of medicine. 2009;360(3):225-35. Epub 2009/01/16. doi: 10.1056/NEJMoa0806994. PubMed PMID: 19144938; PubMed Central PMCID: PMCPMC2713724.

2. Wang PS, Schneeweiss S, Setoguchi S, Patrick A, Avorn J, Mogun H, et al. Ventricular arrhythmias and cerebrovascular events in the elderly using conventional and atypical antipsychotic medications. J Clin Psychopharmacol. 2007;27(6). WOS:000251181600024.

Ventricular Arrhythmia / Sudden Cardiac Death - Excluded

1. Honkola J, Hookana E, Malinen S, Kaikkonen KS, Junttila MJ, Isohanni M, et al. Psychotropic medications and the risk of sudden cardiac death during an acute coronary event. Eur Heart J. 2012;33(6):745-51. Epub 2011/09/17. doi: 10.1093/eurheartj/ehr368. PubMed PMID: 21920969.

2. Jolly K, Gammage MD, Cheng KK, Bradburn P, Banting MV, Langman MJ. Sudden death in patients receiving drugs tending to prolong the QT interval. British journal of clinical pharmacology. 2009;68(5):743-51. Epub 2009/11/18. doi: 10.1111/j.1365-2125.2009.03496.x. PubMed PMID: 19916999; PubMed Central PMCID: PMCPMC2791981.

3. Liperoti R, Gambassi G, Lapane KL, Chiang C, Pedone C, Mor V, et al. Conventional and atypical antipsychotics and the risk of hospitalization for ventricular arrhythmias or cardiac arrest. Archives of internal medicine. 2005;165(6):696-701. Epub 2005/03/30. doi: 10.1001/archinte.165.6.696. PubMed PMID: 15795349.

4. Mehta S, Chen H, Johnson M, Aparasu RR. Risk of serious cardiac events in older adults using antipsychotic agents. The American journal of geriatric pharmacotherapy. 2011;9(2):120-32. Epub 2011/05/14. doi: 10.1016/j.amjopharm.2011.03.004. PubMed PMID: 21565711.

Venous Thromboembolism - Included

1. Liperoti R, Pedone C, Lapane KL, Mor V, Bernabei R, Gambassi G. Venous thromboembolism among elderly patients treated with atypical and conventional antipsychotic agents. Archives of internal medicine. 2005;165(22):2677-82. Epub 2005/12/14. doi: 10.1001/archinte.165.22.2677. PubMed PMID: 16344428.

Venous Thromboembolism - Excluded

1. Allenet B, Schmidlin S, Genty C, Bosson JL. Antipsychotic drugs and risk of pulmonary embolism. Pharmacoepidemiol Drug Saf. 2012;21(1):42-8. Epub 2011/11/05. doi: 10.1002/pds.2210. PubMed PMID: 22052683.

2. Hagg S, Bate A, Stahl M, Spigset O. Associations between venous thromboembolism and antipsychotics - A study of the WHO database of adverse drug reactions. Drug Saf. 2008;31(8). WOS:000258684600005.

3. Jonsson AK, Brudin L, Ahlner J, Hedenmalm K, Eriksson A, Hagg S. Antipsychotics associated with pulmonary embolism in a Swedish medicolegal autopsy series. International clinical psychopharmacology. 2008;23(5). PubMed PMID: MEDLINE:18703935.

4. Jonsson AK, Horvath-Puho E, Hagg S, Pedersen L, Sorensen HT. Antipsychotics and risk of venous thromboembolism: A population-based case-control study. Clinical epidemiology. 2009;1:19-26. Epub 2009/01/01. PubMed PMID: 20865083; PubMed Central PMCID: PMCPMC2943162.

5. Kleijer BC, Heerdink ER, Egberts TC, Jansen PA, van Marum RJ. Antipsychotic drug use and the risk of venous thromboembolism in elderly patients. J Clin Psychopharmacol. 2010;30(5):526-30. Epub 2010/09/04. doi: 10.1097/JCP.0b013e3181f0e87d. PubMed PMID: 20814323.

6. Lacut K, Le Gal G, Couturaud F, Cornily G, Leroyer C, Mottier D, et al. Association between antipsychotic drugs, antidepressant drugs and venous thromboembolism: results from the EDITH case-control study. Fundamental & clinical pharmacology. 2007;21(6):643-

50. Epub 2007/11/24. doi: 10.1111/j.1472-8206.2007.00515.x. PubMed PMID: 18034665.

7. Mehta S, Chen H, Johnson M, Aparasu RR. Risk of serious cardiac events in older adults using antipsychotic agents. The American journal of geriatric pharmacotherapy. 2011;9(2):120-32. Epub 2011/05/14. doi: 10.1016/j.amjopharm.2011.03.004. PubMed PMID: 21565711.

8. Parker C, Coupland C, Hippisley-Cox J. Antipsychotic drugs and risk of venous thromboembolism: nested case-control study. BMJ (Clinical research ed). 2010;341:c4245. Epub 2010/09/23. doi: 10.1136/bmj.c4245. PubMed PMID: 20858909.

Myocardial Infarction - Included

1. Huybrechts KF, Schneeweiss S, Gerhard T, Olfson M, Avorn J, Levin R, et al. Comparative safety of antipsychotic medications in nursing home residents. Journal of the American Geriatrics Society. 2012;60(3):420-9. Epub 2012/02/15. doi: 10.1111/j.1532-5415.2011.03853.x. PubMed PMID: 22329464; PubMed Central PMCID: PMCPMC3302976.

2. Wang PS, Schneeweiss S, Setoguchi S, Patrick A, Avorn J, Mogun H, et al. Ventricular arrhythmias and cerebrovascular events in the elderly using conventional and atypical antipsychotic medications. J Clin Psychopharmacol. 2007;27(6). WOS:000251181600024.

Myocardial Infarction - Excluded

1. Kleijer BC, Koek HL, van Marum RJ, Jansen PA, Egberts TC, Heerdink ER. Risk of acute coronary syndrome in elderly users of antipsychotic drugs: a nested case-control study. Heart (British Cardiac Society). 2012;98(15):1166-71. Epub 2012/06/13. doi: 10.1136/heartjnl-2012-301801. PubMed PMID: 22689716.

2. Mehta S, Chen H, Johnson M, Aparasu RR. Risk of serious cardiac events in older adults using antipsychotic agents. The American journal of geriatric pharmacotherapy. 2011;9(2):120-32. Epub 2011/05/14. doi: 10.1016/j.amjopharm.2011.03.004. PubMed PMID: 21565711.

3. Nakagawa S, Pedersen L, Olsen ML, Mortensen PB, Sorensen HT, Johnsen SP. Antipsychotics and risk of first-time hospitalization for myocardial infarction: a population-based case-control study. Journal of internal medicine. 2006;260(5):451-8. Epub 2006/10/17. doi: 10.1111/j.1365-2796.2006.01708.x. PubMed PMID: 17040251.

Pneumonia - Included

1. Huybrechts KF, Rothman KJ, Silliman RA, Brookhart MA, Schneeweiss S. Risk of death and hospital admission for medical events after initiation of psychotropic medications in older adults admitted to nursing homes. CMAJ. 2011;183(7):E411-9. Epub 2011/03/30. doi: 10.1503/cmaj.101406. PubMed PMID: 21444611; PubMed Central PMCID: PMCPMC3080558.

2. Huybrechts KF, Schneeweiss S, Gerhard T, Olfson M, Avorn J, Levin R, et al. Comparative safety of antipsychotic medications in nursing home residents. Journal of the American Geriatrics Society. 2012;60(3):420-9. Epub 2012/02/15. doi: 10.1111/j.1532-5415.2011.03853.x. PubMed PMID: 22329464; PubMed Central PMCID: PMCPMC3302976.

3. Wang PS, Schneeweiss S, Setoguchi S, Patrick A, Avorn J, Mogun H, et al. Ventricular arrhythmias and cerebrovascular events in the elderly using conventional and atypical antipsychotic medications. J Clin Psychopharmacol. 2007;27(6). WOS:000251181600024.

Pneumonia - Excluded

1. Knol W, van Marum RJ, Jansen PA, Souverein PC, Schobben AF, Egberts AC. Antipsychotic drug use and risk of pneumonia in elderly people. Journal of the American Geriatrics Society. 2008;56(4):661-6. Epub 2008/02/13. doi: 10.1111/j.1532-5415.2007.01625.x. PubMed PMID: 18266664.
2. Pratt N, Roughead EE, Ramsay E, Salter A, Ryan P. Risk of hospitalization for hip fracture and pneumonia associated with antipsychotic prescribing in the elderly: a self-controlled case-series analysis in an Australian health care claims database. Drug safety. 2011;34(7):567-75. Epub 2011/06/15. doi: 10.2165/11588470-000000000-00000. PubMed PMID: 21663332.
3. Star K, Bate A, Meyboom RH, Edwards IR. Pneumonia following antipsychotic prescriptions in electronic health records: a patient safety concern? The British journal of general practice : the journal of the Royal College of General Practitioners. 2010;60(579):e385-94. Epub 2010/10/05. doi: 10.3399/bjgp10X532396. PubMed PMID: 20883613; PubMed Central PMCID: PMCPMC2944948.
4. Trifiro G, Gambassi G, Sen EF, Caputi AP, Bagnardi V, Brea J, et al. Association of community-acquired pneumonia with antipsychotic drug use in elderly patients: a nested case-control study. Annals of internal medicine. 2010;152(7):418-25, W139-40. Epub 2010/04/07. doi: 10.1059/0003-4819-152-7-201004060-00006. PubMed PMID: 20368647.

Hip fracture - Included

1. Huybrechts KF, Rothman KJ, Silliman RA, Brookhart MA, Schneeweiss S. Risk of death and hospital admission for medical events after initiation of psychotropic medications in older adults admitted to nursing homes. CMAJ. 2011;183(7):E411-9. Epub 2011/03/30. doi: 10.1503/cmaj.101406. PubMed PMID: 21444611; PubMed Central PMCID: PMCPMC3080558.
2. Huybrechts KF, Schneeweiss S, Gerhard T, Olfson M, Avorn J, Levin R, et al. Comparative safety of antipsychotic medications in nursing home residents. Journal of the American Geriatrics Society. 2012;60(3):420-9. Epub 2012/02/15. doi: 10.1111/j.1532-5415.2011.03853.x. PubMed PMID: 22329464; PubMed Central PMCID: PMCPMC3302976.

Hip fracture - Excluded

1. Bolton JM, Metge C, Lix L, Prior H, Sareen J, Leslie WD. Fracture risk from psychotropic medications: a population-based analysis. J Clin Psychopharmacol. 2008;28(4):384-91. Epub 2008/07/16. doi: 10.1097/JCP.0b013e31817d5943. PubMed PMID: 18626264.
2. Hien le TT, Cumming RG, Cameron ID, Chen JS, Lord SR, March LM, et al. Atypical antipsychotic medications and risk of falls in residents of aged care facilities. Journal of the American Geriatrics Society. 2005;53(8):1290-5. Epub 2005/08/05. doi: 10.1111/j.1532-5415.2005.53403.x. PubMed PMID: 16078953.

3. Jalbert JJ, Eaton CB, Miller SC, Lapane KL. Antipsychotic use and the risk of hip fracture among older adults afflicted with dementia. J Am Med Dir Assoc. 2010;11(2):120-7. Epub 2010/02/10. doi: 10.1016/j.jamda.2009.10.001. PubMed PMID: 20142067.

4. Kallin K, Gustafson Y, Sandman PO, Karlsson S. Drugs and falls in older people in geriatric care settings. Aging clinical and experimental research. 2004;16(4):270-6. Epub 2004/12/04. PubMed PMID: 15575120.

5. Kolanowski A, Fick D, Waller JL, Ahern F. Outcomes of antipsychotic drug use in community-dwelling elders with dementia. Archives of psychiatric nursing. 2006;20(5):217-25. Epub 2006/10/03. doi: 10.1016/j.apnu.2006.04.004. PubMed PMID: 17010825.

6. Landi F, Onder G, Cesari M, Barillaro C, Russo A, Bernabei R. Psychotropic medications and risk for falls among community-dwelling frail older people: an observational study. The journals of gerontology Series A, Biological sciences and medical sciences. 2005;60(5):622-6. Epub 2005/06/24. PubMed PMID: 15972615.

7. Liperoti R, Onder G, Lapane KL, Mor V, Friedman JH, Bernabei R, et al. Conventional or atypical antipsychotics and the risk of femur fracture among elderly patients: results of a case-control study. The Journal of clinical psychiatry. 2007;68(6):929-34. Epub 2007/06/27. PubMed PMID: 17592919.

8. Mehta S, Chen H, Johnson ML, Aparasu RR. Risk of falls and fractures in older adults using antipsychotic agents: a propensity-matched retrospective cohort study. Drugs & aging. 2010;27(10):815-29. Epub 2010/10/05. doi: 10.2165/11537890-000000000-00000. PubMed PMID: 20883062.

9. Pouwels S, van Staa TP, Egberts AC, Leufkens HG, Cooper C, de Vries F. Antipsychotic use and the risk of hip/femur fracture: a population-based case-control study. Osteoporosis international. 2009;20(9):1499-506. Epub 2009/01/22. doi: 10.1007/s00198-008-0826-5. PubMed PMID: 19156348; PubMed Central PMCID: PMCPMC2728222.

10. Pratt N, Roughead EE, Ramsay E, Salter A, Ryan P. Risk of hospitalization for hip fracture and pneumonia associated with antipsychotic prescribing in the elderly: a self-controlled case-series analysis in an Australian health care claims database. Drug safety. 2011;34(7):567-75. Epub 2011/06/15. doi: 10.2165/11588470-000000000-00000. PubMed PMID: 21663332.
