# Supplementary material for: Quantifying the Role of Adverse Events in the Mortality Difference between First and Second-Generation Antipsychotics in Older Adults: Systematic Review and Meta-Synthesis
Source: PLoS One. 2014 Aug 20;9(8):e105376. doi: 10.1371/journal.pone.0105376 (PMC4139353; doi:10.1371/journal.pone.0105376)
Supplement: File S8 — Detailed descriptions of included studies. (PDF) [file pone.0105376.s009.pdf]

## Supporting Information S8. Detailed descriptions of included studies

Table 1 Detailed descriptions of included studies

| Citation | Population & Design | Event definition, Occurrence, and Adjusted Results (95%CI) | Covariates Used to Adjust Results |
|---|---|---|---|
| Wang 2005. NEJM;353(22):2335-41 | Beneficiaries age 65+ enrolled in Medicare a statewide pharmacy assistance benefit program (Pennsylvania, United States). Closed cohort analysis of 9,142 FGA and 13,748 SGA new users with 6 months follow-up over 1994 to 2003. Data extracted from Medicare insurance claims and pharmacy-dispensing data (Pennsylvania Assistance Contract for the Elderly). Exposure analyzed as "intention to treat." | DEFINITION: All-cause mortality; (at 180 days). OCCURRENCE: FGA users 17.9%; SGA users; 14.6%. ADJUSTED RESULTS: FGA vs SGA <40 days HR=1.56 (1.37 to 1.78); 180 days HR=1.37 (1.27 to 1.49), RD=7.3% (2.0 to 12.6); Dementia [Yes] HR=1.29 (1.15 to 1.45), [No] HR=1.45 (1.30 to 1.63); Nursing Home [Yes] HR=1.26 (1.08 to 1.47), [No] HR=1.42 (1.29 to 1.56). | DEMOGRAPHICS/OTHER: Age, sex, race, calendar time; PSYCHIATRIC COMORBIDITIES: Dementia, delirium, mood dx, psychotic dx, other psychiatric dx; CHRONIC DISEASE COMORBIDITIES: Arrhythmias, diabetes, cerebrovascular dx, congestive heart failure, myocardial infarction, angina, other cardiovascular dx, cancer; MEDICATIONS OR MEDICAL PROCEDURES: Medication use was included in diagnoses for chronic conditions; percutaneous transluminal coronary angioplasty, coronary-artery bypass grafting, nitroglycerin use. |
| Gill 2007. Ann Intern Med;146:775-786 | Beneficiaries age 65+ with dementia diagnosis and no diagnosis of schizophrenia (Ontario, Canada). Closed cohort analysis of 14,213 propensity score-matched pairs of new users, followed for up to 180 days over 1997 to 2003. Data extracted from the Canadian Institute for Health Discharge Abstract Database, Ontario Health Insurance Plan, Registered Persons Database). Exposure analyzed as "per-protocol" - censoring patients after a grace period following discontinuation. | DEFINITION: All-cause mortality. OCCURRENCE: Community Dwelling FGA users 3.7% at 30 days; 13.3 at 180 days; SGA users 2.5% at 30 days, 10.7% at 180 days; Long-term care FGA users 5.8% at 30 days, 20.0% at 180 days; SGA users 4.7% at 30 days; 17.8% at 180 days. ADJUSTED RESULTS: FGA vs SGA; Community dwelling 30 days RD=1.1% (0.5 to 1.8), HR=1.55 (1.19 to 2.02); 180 days RD=2.6% (0.5 to 4.5), HR=1.23 (1.00 to 1.50); Long to term care 30 days RD=1.1% (0.3 to 1.9), HR=1.26 (1.04 to 1.53), 180 days RD=2.2% (0.0 to 4.4), HR=1.27 (1.09 to 1.48). | DEMOGRAPHICS/OTHER: Age, sex; CHRONIC DISEASE COMORBIDITIES: Acute myocardial infarction, congestive heart failure, peripheral vascular dx, cerebrovascular dx, chronic pulmonary dx, connective tissue dx, ulcer dx, mild liver dx, diabetes, hemiplegia or paraplegia, renal dx, liver dx, metastatic cancer. |
| Kales 2007. Am J Psychiatry;164(10):1568-1576 | Beneficiaries, age >65 with dementia diagnosis, covered by the Veterans Health Administration (United States). Closed cohort analysis of 353 FGA and 3,999 SGA new users, followed for 1 year over 2001 to 2005. Data extracted from the Department of Veterans Affairs registries. Exposure analyzed as "intention to treat." | DEFINITION: All-cause mortality. OCCURRENCE: (at 1 year) FGA users 25.2%; SGA users 22.6%. ADJUSTED RESULTS: SGA vs FGA 1 year HR=0.93 (0.75 to 1.16) | DEMOGRAPHICS/OTHER: Age, sex, race, days hospitalized; PSYCHIATRIC COMORBIDITIES: Schizophrenia, bipolar dx, delirium; CHRONIC DISEASE COMORBIDITIES: Charlson comorbidity score, myocardial infarction, congestive heart failure, cerebrovascular dx, chronic pulmonary dx, chronic renal dx, metastatic solid tumor, cancer dx; MEDICATIONS OR MEDICAL PROCEDURES: Cholinesterase inhibitors. |
| Schneeweiss 2007. CMAJ;176(5):627-632 | Persons age 65+ (British Columbia, Canada). Closed cohort analysis of 12,882 FGA and 24,359 SGA new users, followed for 6 months over 1996 to 2004. Data extracted from administrative data from the British Columbia of Health, PharmaNet database, and Vital Statistics Agency. Exposure analyzed as "intention to treat." | DEFINITION: All-cause mortality. OCCURRENCE: FGA users 14.1% at 180 days 31.3 per 100 PY over 5,816 PY; SGA users 9.6% at 180 days 20.6 per 100 PY over 11,354 PY. ADJUSTED RESULTS: FGA vs SGA <40 days HR=1.60 (1.42 to 1.80); 180 days HR=1.32 (1.23 to 1.42), RD=3.5% (2.7 to 4.3); Dementia [Yes] HR=1.26 (1.01 to 1.56), [No] HR=1.30 (1.21 to 1.40); Nursing Home [Yes] HR=1.25 (1.12 to 1.40) [No] HR=1.35 (1.23 to 1.49). | DEMOGRAPHICS/OTHER: Age, sex, race, nursing home residence, calendar time; PSYCHIATRIC COMORBIDITIES: Dementia, delirium, mood dx, psychotic dx, other psychiatric dx; CHRONIC DISEASE COMORBIDITIES: Charlson comorbidity score, arrhythmias, diabetes, cerebral, cerebrovascular dx, angina ,other cardiovascular conditions; MEDICATIONS OR MEDICAL PROCEDURES: anticholinergic rx, percutaneous coronary intervention or coronary artery bypass graft surgery; HEALTH SERVICE: Hospital admission, # physician visits, # distinct prescription rx. |
| Liperoti 2009. J Clin Psychiatry;70(10):1340-1347 | Residents of Medicaid/Medicare-certified nursing homes, age 65+ with dementia (Kansas, Maine, Mississippi, Ohio, South Dakota; United States). Closed cohort analysis of 3,205 FGA and 6,524 SGA new users, followed for 6 months over 1998 to 2000. Baseline, follow-up and administrative data extracted from the Systematic Assessment of Geriatric Drug Use via Epidemiology database and Minimum Data Set. Exposure analyzed as "intention to treat." | DEFINITION: All-cause mortality. OCCURRENCE: FGA users 54.3 per 100 PY; SGA users 40.0 per 100 PY. ADJUSTED RESULTS: FGA vs SGA All nursing home residents 180 days HR=1.41 (1.13 to 1.42); Dementia [Yes] 180 days HR=1.02 (0.75 to 1.39), [No] 180 days HR=1.31 (1.14 to 1.50); Haloperidol vs Risperidone 180 days HR=1.31 (1.13 to 1.53); Phenothiazines vs Risperidone 180 days HR=1.17 (1.00 to 1.53). | DEMOGRAPHICS/OTHER: Age, sex, race, functional impairment, cognitive deficit; PSYCHIATRIC COMORBIDITIES: Alzheimer's dx, Behavioral and psychosocial symptoms of dementia; CHRONIC DISEASE COMORBIDITIES: Hypertension, heart failure, ischemic heart dx, cardiac arrhythmias, diabetes, stroke, COPD, Parkinson's dx; MEDICATIONS OR MEDICAL PROCEDURES: Cardiovascular rx, aspirin/antiplatelets and anticoagulants, benzodiazepines, antidepressants. |

Table 1 Detailed descriptions of included studies

| Citation | Population & Design | Event definition, Occurrence, and Adjusted Results (95%CI) | Covariates Used to Adjust Results |
|---|---|---|---|
| Pratt 2010. Pharmacoepidemiol Drug Saf;19(7):699-707 | Beneficiaries age 65+ covered by the Department of Veterans' Affairs (Australia). Closed cohort analysis of 9,312 FGA and 7,227 SGA new users followed for 1 year over 2003 to 2006. Data extracted from administrative data from the Department of Veterans' Affairs (all medical claims, pharmacy dispensing, and vital status). Exposure analyzed as "intention to treat." | DEFINITION: All-cause mortality; OCCURRENCE: FGA users (at 1 year) 46.8% for all patients (50.4% for nursing home residents); SGA users 29.5% for all patients (37.4% for nursing home residents). ADJUSTED RESULTS: 1 year [All patients] traditional multivariable analysis RD=10.6 (9.2 to 12.1), Instrumental Variable (IV) based on doctor preference RD=23.8 (17.6 to 30.0); [Nursing home residents] traditional multivariable analysis RD=8.5 (6.2 to 10.7), IV analysis based on facility preference RD=10.1 (6.6 to 13.7). | DEMOGRAPHICS/OTHER: Age, sex, residential aged care status; among nursing home residents: type of care, admission type; CHRONIC DISEASE COMORBIDITIES: Stroke, hip fracture, pneumonia, myocardial infarction, chronic heart failure; MEDICATIONS OR MEDICAL PROCEDURES: Morphine, antiepilepsy rx, corticosteroids, NSAIDs, sedative hypnotics, cardiac rx, lipid-lowering rx, calcium channel blockers, anticholinesterase inhibitors, antidepressants, diabetes rx |
| Rossom 2010. J Am Geriatr Soc. 58:1027-2010 | Patients age 65+ with dementia (Texas, United States). Closed cohort analysis of 2,217 haloperidol, 3,384 Olanzapine, and 4,277 Quetiapine (new) users, followed for up to 5 years, over 1999 to 2005. Data extracted from medical and pharmacy records in the Veterans Health Administration National Patient Care Database, Pharmacy Benefits Management Strategic Healthcare Group database. Exposure analyzed as "per protocol" - censoring at discontinuation or initiation of comparator antipsychotic. | DEFINITION: All-cause mortality. OCCURRENCE: (at 30 days) Non-User 1.7 to 2.0%, Haloperidol 5.4%, Olanzapine 2.7%, Quetiapine 1.7%, Risperidone 2.8%; ADJUSTED RESULTS: Compared to Non to users (reference) Haloperidol HR=2.2 (1.7 to 2.9), Olanzapine HR=1.3 (1.0 to 1.7), Quetiapine HR=0.8 , 0.6 to 1.1), Risperidone HR=1.2 (1.0 to 1.4); Compared to Haloperidol (reference) vs Olanzapine HR=1.69 (1.1 to 1.7), Quetiapine HR=2.8 (1.7 to 2.8), Risperidone HR=1.8 (1.4 to 1.7). | DEMOGRAPHICS/OTHER: Age, sex, calendar time (matched); CHRONIC DISEASE COMORBIDITIES: Cerebrovascular dx, ischemic heart dx, peripheral vascular dx, heart failure, cancer, autoimmune deficiency syndrome, diabetes mellitus, COPD, liver dx, renal dx, paraplegia or hemiplegia, rheumatic dx, peptic ulcer dx; MEDICATIONS OR MEDICAL PROCEDURES; HEALTH SERVICE: Acetylcholinesterase inhibitors, alpha-adrenergic receptor blockers, angiotensin-converting enzyme inhibitors, angiotensin receptor blockers, anti-arrhythmics, antibiotics, anticonvulsants, antidepressants, antiplatelet agents, beta-adrenergic receptor blockers, benzodiazepines, calcium channel blockers, cancer chemotherapy, digoxin, diuretics, diabetes rx, hyperlipidemia rx, parkinson's dx rx, peptic ulcer dx rx, pulmonary dx, memantine, nitrates, NSAIDs, opiates, other analgesics, vitamin E, warfarin. |
| Huybrechts 2011. Am J Epidemiol;174(9):1089-1099 | Residents of Medicaid/Medicare-certified nursing homes, age 65+ without diagnosis of cancer, schizophrenia, or bipolar disorder (United States). Closed cohort analysis of 7,252 FGA and 74,760 SGA new users, followed for 6 months, over 2001 to 2005. Data extracted from the Online Survey, Certification and Reporting dataset, Medicare Standard Analytic File, Medicaid Analytic Extract, Minimum Data Set, and the Social Security Death Master File. Exposure analyzed as "intention to treat." | DEFINITION: All-cause mortality. OCCURRENCE: FGA 30.1% at 180 days, 76.0 per 100 PY over 2,873 PY; SGA 19.6% at 180 days, 44.52 per 100 PY over 32,836 PY. ADJUSTED RESULTS: Physician preference based Instrumental Variable (IV)-IRD=26.4 (23.8 to 29.0) per 100 PY, HR=1.54 (1.46 to 1.61); Propensity Score (PS) Matched IV-IRD=7.21 (5.75 to 8.68) per 100 PY, HR=1.31 (1.24 to 1.39); PS trimmed IV-IRD=7.79 (6.61 to 8.96) per 100 PY, HR=1.36 (1.31 to 1.42); PS Adj. untrimmed IV-IRD=23.4 (19.8 to 27.0) per 100 PY, HR=1.49 (1.49 to 1.57). | DEMOGRAPHICS/OTHER: Age, sex, race, functional impairment, cognitive impairment, behavioral problems, calendar year; PSYCHIATRIC COMORBIDITIES: Dementia, depression, anxiety, delirium, psychotic dx, delusions; CHRONIC DISEASE COMORBIDITIES: Myocardial infarction, cardiac arrhythmia, ischemic heart dx, hypertension, congestive heart failure, coronary artery dx, other cardiovascular dx, cerebrovascular dx, parkinson's dx, epilepsy, diabetes, obesity; MEDICATIONS OR MEDICAL PROCEDURES: Antidepressant, hypnotic, other psychotropic rx, dementia rx; HEALTH SERVICE: # days hospitalized, # distinct prescriptions; nursing home facility characteristics: # beds, occupancy rate, availability of Alzheimer's and other special care units, staffing levels, ownership type, proportion of residents whose care was paid for by Medicare/Medicaid, proportion of residents bed-bound, proportion on psychoactive rx, number of deficiencies. |
| Huybrechts 2011. CMAJ; 183(7):E411-E419 | Newly admitted nursing home residents, age 65+ (British Columbia, Canada). Closed cohort analysis of 1902 FGA and 1942 typical new users followed for 6 months over 1996 to 2006. Data extracted from administrative data from the British Columbia of Health, PharmaNet database, and Vital Statistics Agency. Exposure analyzed as "per-protocol" - censoring patients 30 days after discontinuation or upon starting other study drug - and as intention to treat. | DEFINITION: Non-cancer mortality. OCCURRENCE: FGA users 38.8 per 100 PY over 438.8 PY; SGA users 27.3 per 100 PY over 790.4 PY. ADJUSTED RESULTS: FGA vs SGA Per-Protocol HR=1.47 (1.14 to 1.91); Dementia [Yes] HR=1.37 (0.96 to 1.95) [No] HR=1.61 (1.10 to 2.36); Intention to treat HR=1.33 (1.07 to 1.61); Dementia [Yes] HR=1.33 (1.04 to 1.72), [No] HR=1.32 (0.99 to 1.76). | DEMOGRAPHICS/OTHER: Age, sex, calendar year; PSYCHIATRIC COMORBIDITIES: Dementia, depression, anxiety, delirium, mood dx, psychotic dx, sleep dx, alcohol or drug abuse, other psychiatric dx; CHRONIC DISEASE COMORBIDITIES: Hypertensive heart and kidney dx, arrhythmias, diabetes rx, cerebrovascular dx, congestive heart failure, acute myocardial infarction, coronary artery dx, other evidence of ischemic heart dx, other cardiovascular conditions, HIV, epilepsy, parkinson's dx, osteoporosis, fracture history, pneumonia, and COPD, Charlson comorbidity score; MEDICATIONS OR MEDICAL PROCEDURES: Prior use of anticholinergic and psychotropic rx; HEALTH SERVICE: Nursing home, # physician visits, # hospital admissions, # distinct prescription rx, prior specialist care. |

Table 1 Detailed descriptions of included studies

| Citation | Population & Design | Event definition, Occurrence, and Adjusted Results (95%CI) | Covariates Used to Adjust Results |
|---|---|---|---|
| Aparasu 2012. Medical Care;50(11): | Nursing home residents age 65+ dually enrolled in Medicare/Medicaid (Texas, Florida, New York, and California; United States). Closed cohort analysis of 3,609 matched pairs of FGA and SGA new users, followed for 6 months, over 2001 to 2003. Data extracted from the Medicare Standard Analytic File and Medicaid Analytic Extract. Exposure analyzed as "intention to treat" and "per-protocol" - censoring patients 30 days after discontinuation or upon starting other study drug. | DEFINITION: All-cause mortality. OCCURRENCE: (at 180 days) FGA 24.1%; SGA 18.4%. ADJUSTED RESULTS: FGA vs SGA 180 days HR=1.41 (1.27 to 1.57); <40 days HR=1.81 (1.49 to 2.18); 40 to 180 days HR=1.24 (1.09 to 1.42). | DEMOGRAPHICS/OTHER: Age, sex, race; PSYCHIATRIC COMORBIDITIES: Dementia, schizophrenia, anxiety dx, conduct dx, mood dx, other psychotic dx; CHRONIC DISEASE COMORBIDITIES: Hypertension, coronary heart dx, congestive heart failure, acute myocardial infarction, cardiac arrhythmias, circulatory dx, thromboembolic dx, diabetes, cerebrovascular dx, hip/femur fracture, COPD, falls, thyroid dx, renal failure, other renal dx, liver dx, gastric dx, ulcers, cancer, cataract, glaucoma, anemia, osteoporosis, RA, back pain, dyslipidemia, obesity, HIV, pneumonia, parkinson dx, endocarditis, alcohol, extrapyramidal sx; MEDICATIONS OR MEDICAL PROCEDURES: Cardiovascular rx, antidiabetics, analgesics, estrogens, antihistamines, antigastric, anticoagulant, corticosteroids, antiasthmatic rx, anti-infective rx, diuretics, anticancer rx, anticholinergics, ophthalmic rx, antithyroid rx, antismoking rx, endocrine and metabolic rx, hypnotics, antidepressants, anticonvulsants, lithium, antianxiety rx, stimulants; HEALTH SERVICE: Hospitalization. |
| Huybrechts 2012. BMJ;344:e977 | Residents of Medicaid/Medicare-certified nursing homes, age 65+ without diagnosis of cancer, schizophrenia, or bipolar disorder (United States). Closed cohort analysis of 5,904 FGA and 5,541 SGA new users, followed for 6 months over 2001 to 2005. Data extracted from the Online Survey, Certification and Reporting dataset, Medicare Standard Analytic File, Medicaid and Medicare claims, the Minimum Data Set, and the National Death Index. Exposure analyzed as "per-protocol" - censoring patients 30 days after discontinuation, upon starting other study drug, or unknown treatment status (hospital admission stay ≥10 days). | DEFINITION/OCCURRENCE (per 100 PY): Non-cancer mortality, Cause-of Death (via ICD 10 codes) for circulatory, cerebrovascular, respiratory and other diseases. FGA 109.1, SGA 57.5 [averaged over individual SGAs presented in table 2]; Circulatory mortality FGA 51.4, SGA 30.0; Cerebrovascular mortality FGA 11.9, SGA 6.3; Respiratory system mortality FGA 18.9, SGA 8.4; Other mortality (%PY) FGA 38.8, SGA 20.8. DEFINITION/ADJUSTED RESULTS: Non cancer mortality Halodoperidol High vs low dose: HR=1.84 (1.38 to 2.43), Med vs low dose: HR=1.40 (1.10 to 1.77); Haloperidol vs Risperidone High dose: HR=1.70 (1.40 to 2.06); Haloperidol vs Risperidone Low dose: HR=1.41 (1.18 to 1.69); Haloperidol vs Risperidone Non cancer mortality HR=2.07 (1.89 to 2.26), Circulatory mortality, HR=1.86 (1.63 to 2.12), Cerebrovascular mortality, HR=2.23 (1.71 to 2.90), Respiratory mortality, HR=2.53 (2.02 to 3.18), Other mortality, HR=2.19 (1.88 to 2.55). | DEMOGRAPHICS/OTHER: Age, sex, race, education, geographical region, urban, functional impairment, cognitive impairment, delusions, behavior problems; PSYCHIATRIC COMORBIDITIES: Dementia, depression, anxiety, delirium, psychotic dx; CHRONIC DISEASE COMORBIDITIES: Arrhythmias, ischaemic heart dx, hypertension, congestive heart failure, cerebrovascular dx, diabetes, parkinson's dx, Charlson comorbidity score; MEDICATIONS OR MEDICAL PROCEDURES: Antidepressants, hypnotic agents, other psychoactive agents, dementia rx; HEALTH SERVICE: # prescription rx, # outpatient visits, # days in hospital. |
| Kales 2012 Am J Psychiatr;169:71-79 | Patients age 65+ with dementia who received outpatient treatment with antipsychotic (United States). Closed cohort analysis of 2,855 haloperidol, 4,716 Olanzapine, 10,651 Quetiapine, and 13,356 Risperidone; followed for 6 months, over 1999 to 2008. Data extracted from U.S. Department of Veterans Affairs registries. Exposure analyzed as "intention to treat" and "per protocol" - censoring at discontinuation or initiation of a comparator antipsychotic. | DEFINITION: All-cause mortality. OCCURRENCE: Death Rate (per 100 PY) ITT: Haloperidol 45.8 over 1,245 PY, ITT: Olanzapine 27.5 over 2,169 PY, ITT: Quetiapine 18.6 over 5,019 PY, ITT: Risperidone 27.1 over 6,169 PY, PP: Haloperidol 41.4 over 711 PY, PP: Olanzapine 24.4 over 1,521 PY PP: Quetiapine 15.2 over 3,484 PY, PP: Risperidone 22.4 over 4,165 PY. ADJUSTED RESULTS: Haloperidol vs Risperidone ITT: HR=1.50 (1.35 to 1.67), PP: HR=1.59 (1.36 to 1.85); Olanzapine vs Risperidone, ITT: HR=1.02 (0.92 to 1.12), PP: HR=1.06 (0.93 to 1.22); Quetiapine vs Risperidone ITT: HR=0.95 (0.82 to 1.10), PP: HR=0.74 (0.65 to 0.83). | DEMOGRAPHICS/OTHER: Age, sex, race, days hospitalized; PSYCHIATRIC COMORBIDITIES: Schizophrenia, bipolar dx, delirium; CHRONIC DISEASE COMORBIDITIES: Charlson comorbidity score, myocardial infarction, congestive heart failure, cerebrovascular dx, chronic pulmonary dx, chronic renal dx, metastatic solid tumor, cancer dx; MEDICATIONS OR MEDICAL PROCEDURES: Cholinesterase inhibitors. |
| | | Stroke | |

Table 1 Detailed descriptions of included studies

| Citation | Population & Design | Event definition, Occurrence, and Adjusted Results (95%CI) | Covariates Used to Adjust Results |
|---|---|---|---|
| Finkel 2005. Int Psychoger; 17(4):617-629 | Patients age 60+ treated for dementia and enrolled in Medicaid (United States). Closed cohort analysis of 1,260 haloperidol and 8,285 SGA new users, followed for 3 months over 1999 to 2002. Data extracted from Medicaid claims data. Exposure analyzed as "intent to treat." | DEFINITION: Hospitalization admission for cerebrovascular event (430-432, 434-436, 437.1). OCCURRENCE: (at 3 months) Haloperidol 1.19%, Olanzapine 0.96%, Quetiapine 0.56%, Risperidone 0.87%. ADJUSTED RESULTS: Haloperidol vs Risperidone OR=1.91 (1.02 to 3.60), Olanzapine vs Risperidone, OR=1.05 (0.63 to 1.73), Quetiapine vs Risperidone,OR=0.66 (0.23 to 1.87). | DEMOGRAPHICS/OTHER: Age, sex, race; PSYCHIATRIC COMORBIDITIES: Vascular dementia; CHRONIC DISEASE COMORBIDITIES: Prior stroke, hypertension, atherosclerosis, atrial fibrillation, diabetes, hypercholesteremia, carotid artery occlusion; MEDICATIONS OR MEDICAL PROCEDURES: Anticlotting rx; HEALTH SERVICE: Days in hospital, %days study rx available in post index period. |
| Gill 2005. BMJ;330(7489):445 | Persons with dementia diagnosis, age 65+ (Ontario, Canada). Closed cohort analysis of 14,865 FGA and 17,845 SGA new users, with variable follow up over 1997-2002. Data extracted from the Ontario Health Insurance Plan). Exposure analyzed as "per protocol" - censoring until discontinuation or initiating a comparator antipsychotic. | DEFINITION: Hospital admission for ischaemic stroke (ICD-9 codes 431,432, 436). OCCURRENCE: FGA users 1.5% at 2 years, 2.23 per 100 PY over average 250 days; SGA users 1.6% at 2 years, 2.55 per 100 PY over average 223 days. ADJUSTED RESULTS: FGA vs SGA [Full cohort] HR=1.01 (0.81 to 1.26), [History of Stroke] HR=0.80 (0.55 to 1.16) [Long to term care resident] HR=1.15 (0.82 to 1.60) [Users with ≥2 dispensings] HR=0.89 (0.69 to 1.17) [History of Atrial fibrillation] HR=1.23 (0.70 to 2.02). | DEMOGRAPHICS/OTHER: Age, sex, low income, residence in long-term care; CHRONIC DISEASE COMORBIDITIES: Prior stroke, atrial fibrillation, diabetes mellitus, acute myocardial infarction, congestive heart failure; MEDICATIONS OR MEDICAL PROCEDURES: # distinct rx, antihypertensives, angiotensin converting enzyme inhibitors, lipid lowering rx, antidiabetic rx, hormone replacement therapies; HEALTH SERVICE: # physician claims per year. |
| Wang 2007. Journal of clinical psychopharmacology; 27(6):707-10 | Beneficiaries age 65+ enrolled in Medicare a statewide pharmacy assistance benefit program (Pennsylvania, United States). Closed cohort analysis of 9,142 FGA and 13,748 SGA new users with 6 months follow-up over 1994 to 2003. Data extracted from Medicare insurance claims and pharmacy-dispensing data (Pennsylvania Assistance Contract for the Elderly). Exposure analyzed as "intention to treat." | DEFINITION: Cerebrovascular event (diagnosis). OCCURRENCE: Not reported. ADJUSTED RESULTS: FGA vs SGA 30 days HR=1.08 (0.99 to 1.18); 60 days, HR=1.10 (1.02 to 1.19); 120 days HR=1.09 (1.02 to 1.16). | DEMOGRAPHICS/OTHER: Age, sex, race; PSYCHIATRIC COMORBIDITIES: Dementia, delirium, mood dx, psychotic dx, and other psychiatric dx; CHRONIC DISEASE COMORBIDITIES: Acute myocardial infarction, cardiac arrhythmia, cerebrovascular events, congestive heart failure, pneumonia, other serious bacterial infections, diabetes, ischemic heart dx, other cardiovascular conditions, cancers, HIV; MEDICATIONS OR MEDICAL PROCEDURES: Medication sometimes used in definitions of chronic conditions; # psychiatric rx use, # total rx; HEALTH SERVICE: Hospitalizations, nursing home stays. |
| Sacchetti 2008. J Psychopharmacol;22(1):39 | Primary care patients age 65+ without diagnosis of cerebral tumors, coagulopathy, history or diagnosis of stroke (Italy). Closed cohort analysis of 1,656 conventional (749 butyrophenone and 907 phenothiazine) and 599 atypical new users followed for up to 3 years (87 to 112 days on average) from 2000 to 2003. Data extracted from the Health Search Database. Exposure analyzed as "per protocol" - censoring at 30 days after discontinuation. | DEFINITION: Incident stroke (ICD-9 codes 434.9, 438.0, 362, 342.0, 342.1, and 342.9 or encoded medical problems described as stroke, hemiparesis or hemiplegia). OCCURRENCE: Butryphenones 4.71 per 100 PY, Phenothiazines 7.27 per 100 PY, SGA 4.74 per 100 PY. ADJUSTED RESULTS: Compared to SGA users Butyrophenones HR=1.44 (0.55 to 3.76) Phenothiazines HR=2.34 (1.01 to 5.41). | DEMOGRAPHICS/OTHER: Age, sex, race; PSYCHIATRIC COMORBIDITIES: Dementia, psychiatric indication for antipsychotic use; CHRONIC DISEASE COMORBIDITIES: Chronic dx score, Parkinson's dx, hypertension, ischemic heart dx, heart failure, atrial fibrillation, diabetes, dyslipidemia, COPD, recent history of pneumonia, malignant neoplasm, obesity; MEDICATIONS OR MEDICAL PROCEDURES: Benzodiazepines, diuretics, CCBs, ACE-inhibitors, beta-blockers, anticoagulants, angiotensin receptor blockers, sympathicomimetic rx. |
| Chan 2010. Int J Geriatr Psychiatry;25(4):362-70 | Dementia patients age 65+ receiving their 1st psychiatric service at an acute regional hospital (Hong Kong). Closed cohort analysis of 654 conventional users, 72 atypical new users, and 363 non-users, followed for an average 628-1019 days, over 2000 to 2007. Data extracted from administrative records from a large, acute, regional hospital. Exposure analyzed as "per protocol" - censoring until discontinuation or initiating a comparator antipsychotic. | DEFINITION: Hospital admission for cerebrovascular adverse event as primary diagnosis (including subarachnoid, intracerebra, or cranial haemorrhage, cerebral occlusion, stenosis, thrombosis, transient ischemic attack, others). OCCURRENCE: FGA users 3.27 per 100 PY over median 1019 days; SGA users 4.96 per 100 PY over median 697 days; Non-users 4.46 per 100 PY over median 628 days. ADJUSTED RESULTS: Compared to Non-users FGA HR=0.964 (.584 to 1.591), SGA HR=1.036 (0.350 to 3.066); FGA vs SGA HR=1.03 (0.51 to 1.67). | DEMOGRAPHICS/OTHER: Age, sex, marital status, education level, residency, smoking; PSYCHIATRIC COMORBIDITIES: Dementia type; CHRONIC DISEASE COMORBIDITIES: Prior stroke, atrial fibrillation, hypertension, diabetes, hypercholesterolaemia, ischaemic heart dx, congestive heart dx, rheumatic heart dx, malignant neoplasm; MEDICATIONS OR MEDICAL PROCEDURES: Antipsychotics, antidepressants, anxiolytics; HEALTH SERVICE: # rx prescription rx. |

Table 1 Detailed descriptions of included studies

| Citation | Population & Design | Event definition, Occurrence, and Adjusted Results (95%CI) | Covariates Used to Adjust Results |
|---|---|---|---|
| Huybrechts 2012. J Am Geriatr Soc 60:420-429 | Residents of Medicaid/Medicare-certified nursing homes, age 65+ without diagnosis of cancer, schizophrenia, or bipolar disorder (United States). Closed cohort analysis of 7,463 FGA and 76,496 SGA new users, followed for 6 months, over 2001 to 2005. Data extracted from the Online Survey, Certification and Reporting dataset, Medicare Standard Analytic File, Medicaid Analytic Extract, Minimum Data Set, and the Social Security Death Master File. Exposure analyzed as "per protocol" - censoring at discontinuation or initiation of comparator antipsychotic; also censored at any hospitalization. | DEFINITION: Hospitalization for cerebrovascular event, stroke or transient ischemic attack (ICD-9 codes 430-438). OUTCOME: FGA 10.2 per 100 PY; SGA 9.2 per 100 PY. ADJUSTED RESULTS: FGA vs SGA HR=0.81 (0.65 to 1.01). | DEMOGRAPHICS/OTHER: Age, sex race, education, and state of residence, cognitive impairment, functional impairment; PSYCHIATRIC COMORBIDITIES: Dementia, depression, anxiety, delirium, psychotic dx; CHRONIC DISEASE COMORBIDITIES: Myocardial infarction, arrhythmia, ischemic heart dx, hypertension, congestive heart failure, cerebrovascular dx, diabetes, Parkinson's dx; MEDICATIONS OR MEDICAL PROCEDURES: Antidepressants, hypnotic agents, other psychoactive agents, dementia rx; HEALTH SERVICE: # rx prescription rx, # outpatient visits, # hospital days. |
| | | Ventricular Arrhythmia / Sudden Cardiac Death | |
| Wang 2007. Journal of clinical psychopharmacology; 27(6):707-10 | Beneficiaries age 65+ enrolled in Medicare a statewide pharmacy assistance benefit program (Pennsylvania, United States). Closed cohort analysis of 9,142 FGA and 13,748 SGA new users with 6 months follow-up over 1994 to 2003. Data extracted from Medicare insurance claims and pharmacy-dispensing data (Pennsylvania Assistance Contract for the Elderly). Exposure analyzed as "intention to treat." | DEFINITION: Ventricular arrhythmia diagnosis plus use of group I-IV antiarrhythmia medication. OCCURRENCE: Not provided. ADJUSTED RESULTS: FGA vs SGA 30 days HR=1.20 (1.03 to 1.39); 60 days HR=1.10 (0.98 to 1.24); 120 days HR=1.06 (0.96 to 1.17). | DEMOGRAPHICS/OTHER: Age, sex, race; PSYCHIATRIC COMORBIDITIES: Dementia, delirium, mood dx, psychotic dx, and other psychiatric dx; CHRONIC DISEASE COMORBIDITIES: Acute myocardial infarction, cardiac arrhythmia, cerebrovascular events, congestive heart failure, pneumonia, other serious bacterial infections, diabetes, ischemic heart dx, other cardiovascular conditions, cancers, HIV; MEDICATIONS OR MEDICAL PROCEDURES: Medication sometimes used in definitions of chronic conditions; # psychiatric rx use, # total rx; HEALTH SERVICE: Hospitalizations, nursing home stays. |
| Ray 2009. N Engl J Med;360(3):225 | Persons age 30-74 (mean 45.7), excluding patients at high risk for non-cardiac causes of death (Tennessee, United States). Open cohort analysis of 44,218 conventional and 46,089 atypical users and 186,600 non-users, matched on age, sex (or propensity score). Followed over 1990 to 2005. Data extracted from Medicaid claims and death certificates. Exposure analyzed "as treated." | DEFINITION: Sudden cardiac death occurring in the community. Excluded deaths were that were not sudden, occurred in a hospital, involved a plausible non-cardiac cause, or involved a cardiac cause not consistent with ventricular tachyarrhythmia. OCCURRENCE: Prevalent FGA user 0.29 per 100 PY over 86,735 PY; Prevalent SGA user; 0.28 per 100 PY over 79,589 PY; Non-user 0.14 per 100 PY over 624,591 PY; Prevalent FGA/SGA users age 70-74; 0.476 per 100 PY. ADJUSTED RESULTS: Current new users [all ages] FGA vs non to user IRR=1.74 (1.14 to 2.67); SGA vs non to user IRR=1.86 (1.35 to 2.57); FGA vs SGA ratio of ratio estimate IRR=0.94 (0.84 to 1.04). | DEMOGRAPHICS/OTHER: age, sex, race, urban residence, Medicaid enrollment due to disability; PSYCHIATRIC COMORBIDITIES: Schizophrenia and other psychoses, mood dx, organic mental dx, dementia, alcohol, prescription drug dependence, history of convulsions or seizure disorder, and psychiatric health care utilization; CHRONIC DISEASE COMORBIDITIES: Prior revascularization, myocardial infarction or other coronary heart dx, heart failure, conduction dx or arrhythmia, valve dx, cerebrovascular dx, peripheral vascular dx, hypertension, hyperlipidemia, renal failure, obesity, smoking-related dx, COPD; MEDICATIONS OR MEDICAL PROCEDURES: Anti-arrhythmics, angiotensin converting-enzyme inhibitors and angiotensin receptor blockers, anticoagulants, antidiabetic rx, aspirin, non-aspirin anti-platelet agents, beta-blockers, calcium channel blockers, digoxin and other inotropic agents, statins, other lipid-lowering agents, loop diuretics, thiazide and other diuretics,, nitrates, other antihypertensive rx, and pentoxifylline/related rx; HEALTH SERVICE: Compliance index, psychiatric hospitalization, any hospitalization, emergency department visit. |
| | | Venous Thromboembolism | |

Table 1 Detailed descriptions of included studies

| Citation | Population & Design | Event definition, Occurrence, and Adjusted Results (95%CI) | Covariates Used to Adjust Results |
|---|---|---|---|
| Liperoti 2005. Arch Intern Med;165(22):2677 | Medicare-certified nursing home residents age 65+ without schizophrenia (United States). Closed cohort analysis of 19,940 new users and 112,078 non-users, followed for 6 months over 1998 to 1999. Baseline, follow-up and administrative data extracted from the Systematic Assessment of Geriatric Drug Use via Epidemiology database and Minimum Data Set. Exposure analyzed as "intention to treat." | DEFINITION: First hospitalization for Venous thromboembolism (VTE) (ICD-9 codes 453.8, 451.11, 451.19, 451.81, 451.1, 415.1) VTE cases=77.6% VT, 22.4% PE. OCCURRENCE: FGA users 0.84 per 100 PY over 3,318 PY; SGA users 1.24 per 100 PY over 5,173 PY; Non-users 0.87 per 100 PY over 50,604 PY. ADJUSTED RESULTS: Compared to non-users, FGA users HR=1.02 (0.67 to 1.55), SGA users HR=2.01 (1.50 to 2.70); Ratio of ratio estimate FGA vs SGA users HR=0.50 (0.45 to 0.57). Similar results in those without main VTE risk/protective factors, and in those with cognitive impairment. | DEMOGRAPHICS/OTHER: Age, sex, functional status, cognitive status; PSYCHIATRIC COMORBIDITIES: Dementia, depression; CHRONIC DISEASE COMORBIDITIES: BMI, deep vein thrombosis, hip fracture, COPD, cancer, peripheral vascular dx, cerebrovascular dx, heart failure, diabetes; MEDICATIONS OR MEDICAL PROCEDURES: Anticoagulant, aspirin, antiplatelets, estrogens. |
| | | Myocardial Infarction | |
| Wang 2007. Journal of clinical psychopharmacology; 27(6):707-10 | Beneficiaries age 65+ enrolled in Medicare a statewide pharmacy assistance benefit program (Pennsylvania, United States). Closed cohort analysis of 9,142 FGA and 13,748 SGA new users with 6 months follow-up over 1994 to 2003. Data extracted from Medicare insurance claims and pharmacy-dispensing data (Pennsylvania Assistance Contract for the Elderly). Exposure analyzed as "intention to treat." | DEFINITION: Hospitalization for acute myocardial infarction (principal or secondary diagnostic position or diagnosis related group codes). OCCURRENCE: Not reported. ADJUSTED RESULTS: FGA vs SGA 30 days HR=0.89 (0.59 to 1.33); 60 days HR=1.02 (0.75 to 1.40); 120 days HR=1.16 (0.91 to 1.48). | DEMOGRAPHICS/OTHER: Age, sex, race; PSYCHIATRIC COMORBIDITIES: Dementia, delirium, mood dx, psychotic dx, and other psychiatric dx; CHRONIC DISEASE COMORBIDITIES: Acute myocardial infarction, cardiac arrhythmia, cerebrovascular events, congestive heart failure, pneumonia, other serious bacterial infections, diabetes, ischemic heart dx, other cardiovascular conditions, cancers, HIV; MEDICATIONS OR MEDICAL PROCEDURES: Medication sometimes used in definitions of chronic conditions; # psychiatric rx use, # total rx; HEALTH SERVICE: Hospitalizations, nursing home stays. |
| Huybrechts 2012. J Am Geriatr Soc 60:420-429 | Residents of Medicaid/Medicare-certified nursing homes, age 65+ without diagnosis of cancer, schizophrenia, or bipolar disorder (United States). Closed cohort analysis of 7,463 FGA and 76,496 SGA new users, followed for 6 months, over 2001 to 2005. Data extracted from the Online Survey, Certification and Reporting dataset, Medicare Standard Analytic File, Medicaid Analytic Extract, Minimum Data Set, and the Social Security Death Master File. Exposure analyzed as "per protocol" - censoring at discontinuation or initiation of comparator antipsychotic; also censored at any hospitalization | DEFINITION: Hospitalization for myocardial infarction (ICD-9 codes 410 excluding 410.x2 or DRG codes 121-123; with length of stay ≥3 days unless patient dies in-hospital). OCCURRENCE: FGA 3.5 per 100 PY; SGA 2.0 per 100 PY. ADJUSTED RESULTS: FGA vs SGA HR=1.23 (0.82 to 1.82). | DEMOGRAPHICS/OTHER: Age, sex race, education, and state of residence, cognitive impairment, functional impairment; PSYCHIATRIC COMORBIDITIES: Dementia, depression, anxiety, delirium, psychotic dx; CHRONIC DISEASE COMORBIDITIES: Myocardial infarction, arrhythmia, ischemic heart dx, hypertension, congestive heart failure, cerebrovascular dx, diabetes, Parkinson's dx; MEDICATIONS OR MEDICAL PROCEDURES: Antidepressants, hypnotic agents, other psychoactive agents, dementia rx; HEALTH SERVICE: # rx prescription rx, # outpatient visits, # hospital days. |
| | | Pneumonia | |
| Wang 2007. Journal of clinical psychopharmacology; 27(6):707-10 | Beneficiaries age 65+ enrolled in Medicare a statewide pharmacy assistance benefit program (Pennsylvania, United States). Closed cohort analysis of 9,142 FGA and 13,748 SGA new users with 6 months follow-up over 1994 to 2003. Data extracted from Medicare insurance claims and pharmacy-dispensing data (Pennsylvania Assistance Contract for the Elderly). Exposure analyzed as "intention to treat." | DEFINITION: Pneumonia diagnostic codes plus prescription for antibiotic medication. OCCURRENCE: Not reported. ADJUSTED RESULTS: FGA vs SGA 30 days HR=1.11 (0.76 to 1.63); 60 days HR=1.03 (0.76 to 1.38); 120 days HR=0.84 (0.66 to 1.05). | DEMOGRAPHICS/OTHER: Age, sex, race; PSYCHIATRIC COMORBIDITIES: Dementia, delirium, mood dx, psychotic dx, and other psychiatric dx; CHRONIC DISEASE COMORBIDITIES: Acute myocardial infarction, cardiac arrhythmia, cerebrovascular events, congestive heart failure, pneumonia, other serious bacterial infections, diabetes, ischemic heart dx, other cardiovascular conditions, cancers, HIV; MEDICATIONS OR MEDICAL PROCEDURES: Medication sometimes used in definitions of chronic conditions; # psychiatric rx use, # total rx; HEALTH SERVICE: Hospitalizations, nursing home stays. |

Table 1 Detailed descriptions of included studies

| Citation | Population & Design | Event definition, Occurrence, and Adjusted Results (95%CI) | Covariates Used to Adjust Results |
|---|---|---|---|
| Huybrechts 2011. CMAJ; 183(7):E411-E419 | Newly admitted nursing home residents, age 65+ (British Columbia, Canada). Closed cohort analysis of 1902 FGA and 1942 typical new users followed for 6 months over 1996 to 2006. Data extracted from administrative data from the British Columbia of Health, PharmaNet database, and Vital Statistics Agency. Exposure analyzed as "per-protocol" - censoring patients 30 days after discontinuation or upon starting other study drug - and as intention to treat. | DEFINITION: Hospitalization for Pneumonia ICD9 480-486; ICD10 J12-J18. OCCURRENCE: FGA users 9.9 per 100 PY over 431.2 PY; SGA users 7.6 per 100 PY over 790.4 PY. ADJUSTED RESULTS: FGA vs SGA Per-Protocol HR=1.03 (0.62 to 1.69), Intention to treat HR=0.96 (0.66 to 1.41). | DEMOGRAPHICS/OTHER: Age, sex, calendar year; PSYCHIATRIC COMORBIDITIES: Dementia, depression, anxiety, delirium, mood dx, psychotic dx, sleep dx, alcohol or drug abuse, other psychiatric dx; CHRONIC DISEASE COMORBIDITIES: Hypertensive heart and kidney dx, arrhythmias, diabetes rx, cerebrovascular dx, congestive heart failure, acute myocardial infarction, coronary artery dx, other evidence of ischemic heart dx, other cardiovascular conditions, HIV, epilepsy, Parkinson's dx, osteoporosis, fracture history, pneumonia, and COPD, Charlson comorbidity score; MEDICATIONS OR MEDICAL PROCEDURES: Prior use of anticholinergic and psychotropic rx; HEALTH SERVICE: Nursing home, # physician visits, # hospital admissions, # distinct prescription rx, prior specialist care. |
| Huybrechts 2012. J Am Geriatr Soc 60:420-429 | Residents of Medicaid/Medicare-certified nursing homes, age 65+ without diagnosis of cancer, schizophrenia, or bipolar disorder (United States). Closed cohort analysis of 7,463 FGA and 76,496 SGA new users, followed for 6 months, over 2001 to 2005. Data extracted from the Online Survey, Certification and Reporting dataset, Medicare Standard Analytic File, Medicaid Analytic Extract, Minimum Data Set, and the Social Security Death Master File. Exposure analyzed as "per protocol" - censoring at discontinuation or initiation of comparator antipsychotic; also censored at any hospitalization. | DEFINITION: Hospitalization for pneumonia (ICD-9 codes). OCCURRENCE: FGA 3.6 per 100 PY; SGA 1.9 per 100 PY. ADJUSTED RESULTS: FGA vs SGA HR=1.28 (0.87 to 1.88). | DEMOGRAPHICS/OTHER: Age, sex race, education, and state of residence, cognitive impairment, functional impairment; PSYCHIATRIC COMORBIDITIES: Dementia, depression, anxiety, delirium, psychotic dx; CHRONIC DISEASE COMORBIDITIES: Myocardial infarction, arrhythmia, ischemic heart dx, hypertension, congestive heart failure, cerebrovascular dx, diabetes, Parkinson's dx; MEDICATIONS OR MEDICAL PROCEDURES: Antidepressants, hypnotic agents, other psychoactive agents, dementia rx; HEALTH SERVICE: # rx prescription rx, # outpatient visits, # hospital days. |
| | | Hip Fracture | |
| Huybrechts 2011. CMAJ; 183(7):E411-E419 | Newly admitted nursing home residents, age 65+ (British Columbia, Canada). Closed cohort analysis of 1902 FGA and 1942 typical new users followed for 6 months over 1996 to 2006. Data extracted from administrative data from the British Columbia of Health, PharmaNet database, and Vital Statistics Agency. Exposure analyzed as "per-protocol" - censoring patients 30 days after discontinuation or upon starting other study drug - and as intention to treat. | DEFINITION: Hospitalization for Femur fracture hospitalization (ICD-9 codes 820-821, plus related procedure codes and minimum 1 day stay). OCCURRENCE: FGA users 13.0 per 100 PY over 431.2 PY; SGA users 8.5 per 100 PY over 656.0 PY. ADJUSTED RESULTS: FGA vs SGA Per-Protocol HR=1.61 (1.03 to 2.51); Intention to treat HR=1.16 (0.82 to 1.63). | DEMOGRAPHICS/OTHER: Age, sex, calendar year; PSYCHIATRIC COMORBIDITIES: Dementia, depression, anxiety, delirium, mood dx, psychotic dx, sleep dx, alcohol or drug abuse, other psychiatric dx; CHRONIC DISEASE COMORBIDITIES: Hypertensive heart and kidney dx, arrhythmias, diabetes rx, cerebrovascular dx, congestive heart failure, acute myocardial infarction, coronary artery dx, other evidence of ischemic heart dx, other cardiovascular conditions, HIV, epilepsy, parkinson's dx, osteoporosis, fracture history, pneumonia, and COPD, Charlson comorbidity score; MEDICATIONS OR MEDICAL PROCEDURES: Prior use of anticholinergic and psychotropic rx; HEALTH SERVICE: Nursing home, # physician visits, # hospital admissions, # distinct prescription rx, prior specialist care. |
| Huybrechts 2012. J Am Geriatr Soc 60:420-429 | Residents of Medicaid/Medicare-certified nursing homes, age 65+ without diagnosis of cancer, schizophrenia, or bipolar disorder (United States). Closed cohort analysis of 7,463 FGA and 76,496 SGA new users, followed for 6 months, over 2001 to 2005. Data extracted from the Online Survey, Certification and Reporting dataset, Medicare Standard Analytic File, Medicaid Analytic Extract, Minimum Data Set, and the Social Security Death Master File. Exposure analyzed as "per protocol" - censoring at discontinuation or initiation of comparator antipsychotic; also censored at any hospitalization | DEFINITION: Hospitalization for hip fracture with procedural code indicating surgical repair (ICD-9 diagnostic/procedure codes and CPT-4 codes). OCCURRENCE: FGA 5.3 per 100 PY; SGA 3.8 per 100 PY. ADJUSTED RESULTS: FGA vs SGA HR=1.27 (0.94 to 1.72). | DEMOGRAPHICS/OTHER: Age, sex race, education, and state of residence, cognitive impairment, functional impairment; PSYCHIATRIC COMORBIDITIES: Dementia, depression, anxiety, delirium, psychotic dx; CHRONIC DISEASE COMORBIDITIES: Myocardial infarction, arrhythmia, ischemic heart dx, hypertension, congestive heart failure, cerebrovascular dx, diabetes, Parkinson's dx; MEDICATIONS OR MEDICAL PROCEDURES: Antidepressants, hypnotic agents, other psychoactive agents, dementia rx; HEALTH SERVICE: # rx prescription rx, # outpatient visits, # hospital days. |
